# Deep Learning Automates the Quantitative Analysis of Individual Cells in Live-Cell Imaging Experiments

**DOI:** 10.1371/journal.pcbi.1005177

**Published:** 2016-11-04

**Authors:** David A. Van Valen, Takamasa Kudo, Keara M. Lane, Derek N. Macklin, Nicolas T. Quach, Mialy M. DeFelice, Inbal Maayan, Yu Tanouchi, Euan A. Ashley, Markus W. Covert

**Affiliations:** 1 Department of Bioengineering, Stanford University, Stanford, California, United States of America; 2 Department of Chemical and Systems Biology, Stanford University, Stanford, California, United States of America; 3 Department of Genetics, Stanford University, Stanford, California, United States of America; 4 Department of Cardiovascular Medicine, Stanford University, Stanford, California, United States of America; National Institutes of Health, UNITED STATES

## Abstract

Live-cell imaging has opened an exciting window into the role cellular heterogeneity plays in dynamic, living systems. A major critical challenge for this class of experiments is the problem of image segmentation, or determining which parts of a microscope image correspond to which individual cells. Current approaches require many hours of manual curation and depend on approaches that are difficult to share between labs. They are also unable to robustly segment the cytoplasms of mammalian cells. Here, we show that deep convolutional neural networks, a supervised machine learning method, can solve this challenge for multiple cell types across the domains of life. We demonstrate that this approach can robustly segment fluorescent images of cell nuclei as well as phase images of the cytoplasms of individual bacterial and mammalian cells from phase contrast images without the need for a fluorescent cytoplasmic marker. These networks also enable the simultaneous segmentation and identification of different mammalian cell types grown in co-culture. A quantitative comparison with prior methods demonstrates that convolutional neural networks have improved accuracy and lead to a significant reduction in curation time. We relay our experience in designing and optimizing deep convolutional neural networks for this task and outline several design rules that we found led to robust performance. We conclude that deep convolutional neural networks are an accurate method that require less curation time, are generalizable to a multiplicity of cell types, from bacteria to mammalian cells, and expand live-cell imaging capabilities to include multi-cell type systems.

## Author Summary

Dynamic live-cell imaging experiments are a powerful tool to interrogate biological systems with single cell resolution. The key barrier to analyzing data generated by these measurements is image segmentation—identifying which parts of an image belong to which individual cells. Here we show that deep learning is a natural technology to solve this problem for these experiments. We show that deep learning is more accurate, requires less time to curate segmentation results, can segment multiple cell types, and can distinguish between different cell lines present in the same image. We highlight specific design rules that enable us to achieve high segmentation accuracy even with a small number of manually annotated images (~100 cells). We expect that our work will enable new experiments that were previously impossible, as well as reduce the computational barrier for new labs to join the live-cell imaging space.

This is a *PLOS Computational Biology* Methods paper.

## Introduction

Live-cell imaging, in which living cells are imaged over a period of time using phase contrast and/or fluorescence microscopy, is a powerful method for interrogating living systems. This class of experiments has shed light on numerous biological problems, which include transcriptional regulation in both bacteria and eukaryotes as well as information transmission in mammalian signaling networks [1–8]. One common insight these single-cell measurements have been able to provide is the role cellular heterogeneity plays in these systems, as they have the ability to capture the differences between cells and observe how these differences evolve in time. This aspect of living systems is often missed in population level analyses and is best captured by live-cell imaging [9].

Central to the analysis for these experiments is the problem of image segmentation—the assignment of which pixels in an image belong to each individual cell [9]. Without this mapping, it is impossible to extract statistics on cellular fluorescence or geometry with single-cell resolution. The available solutions to date draw upon a standard set of techniques—filtering, morphological operations, thresholding, and the watershed transform—from computer vision. Typically, a combination of these tools are tailored to each individual experiment [4, 10–13]. For example, CellProfiler and Oufti are tools to perform cell segmentation and tracking (primarily using thresholding, watershed transform, and Voronoi algorithms). Supervised machine learning methods have also seen significant success in this space [14–16]. Two notable examples are Ilastik and Microscopy Image Browser, two freely available programs that use supervised machine learning (edge and texture filters with random forest classification) to perform segmentation [12, 17–19].

Our lab’s experience in performing all aspects live-cell imaging experiments—from data generation to analysis—have lead us to identify three main challenges in this space. These are curation time, segmentation accuracy, and solution sharing. The unfortunate reality is that the output of most programs built around the standard set of techniques described above requires significant amounts of manual curation. The specific time cost is rarely, if ever, reported, but work by our lab on NF-κ B signaling in mammalian cells, which required segmenting images of fluorescent nuclei, required on the order of 100+ hours of manual curation per manuscript [4, 5]. Similarly, recently-published work requiring segmentation of growing micro-colonies of *E*. *coli* required ~40 hours [20]. Much of this burden can be traced to inaccurate segmentation algorithms and the time required to separate accurately segmented cells from inaccurately segmented ones. The need for human curation is a significant drawback to these methods; not only are far fewer experiments performed than could be, but many types of experiments are never performed because the analysis is seen as prohibitive (co-culture, for example—see [5]).

The image analysis techniques mentioned above are also confounded by commonly-desired tasks, such as robust segmentation of mammalian cell cytoplasms, or bacterial cells in close proximity. Segmentation methods exist for the mammalian cytoplasm, but they typically require either imaging a cytoplasmic fluorescent protein (which removes a fluorescence channel) or imaging multiple focal planes (which increases acquisition time) [21–26]. Neither of these consequences are desirable. As a result, the cytoplasmic segmentation problem is frequently circumvented by sampling pixels in close proximity to the nucleus and using them as a proxy for the cytoplasm [27–29]. More progress has been made in segmenting closely packed bacterial cells [17]; however, a robust method to identify the cytoplasm of mammalian cells or bacterial micro-colonies with single-cell resolution directly from phase microscopy images has remained elusive [17, 26, 30, 31].

Another challenge concerns generality, or the ability of existing solutions or software in one lab to be applied to the problems of another lab. Because different groups use highly-tuned combinations of these standard techniques to solve the image segmentation problem for specific experiments, there is a barrier to sharing work and ideas in this space. CellProfiler, Oufti, and Ilastik represent notable exceptions, and have empowered a number of experiments, including in labs which were otherwise new to computational image analysis [12, 17, 18]. However, the overall lack of sharable segmentation solutions means the cost of entering this field requires a significant—and often unanticipated—computational investment, beyond the obvious costs associated with the microscopy itself.

Recent advances in supervised machine learning, namely deep convolutional neural networks (referred to here as conv-nets) have shown remarkable performance for the task of image classification—that is, assigning descriptive labels to images [32, 33]. By “learning” on a set of manually annotated data, conv-nets can be used to classify new images with remarkable accuracy [33]. Prior work has shown that in addition to functioning as image classifiers, conv-nets can also perform semantic segmentation—the assignment of class labels to each individual pixel of an image rather than to the whole image itself—in a computationally efficient manner [34–36]. Despite their successful application to numerous real-world problems, conv-nets have only recently been applied to analyzing biological data. Recent work has demonstrated that they have significant potential to address the needs of live cell imaging experiments. Work by Ronnenberg et. al. won the 2015 ISBI cell tracking competition, and was the first to demonstrate that conv-nets can segment images of U373 (with almost perfect accuracy) and HeLa cells from DIC microscopy images [37]. Another recent paper addressed the problem of segmenting fluorescent images of yeast cells [37–40]. Much like Krizhevsky’s initial application of conv-nets to the ImageNet image classification challenge in 2012, this work strongly suggests that conv-nets are a natural technology for live-cell imaging experiments [33]. While these recent developments have the potential to be revolutionary, our experience both as experimentalists and as users of deep learning demonstrated that there was still work to be done before conv-nets can supplant prior image segmentation methods in this space. In this work, we build on these exciting advances to demonstrate that conv-nets address all of the image analysis problems described above for a large variety of cell types. Our aim was to leverage our lab’s experience in live-cell imaging and deep learning to develop a robust tool that could be applied to a variety of live-cell imaging experiments.

Our paper is organized as follows. We first review how image segmentation can be thought of as image classification as well as the mathematical structure of conv-nets. We show that conv-nets can accurately segment the cytoplasms of bacterial cells and mammalian cell nuclei from fluorescent microscopy images. We show that integrating phase microscopy images with fluorescent microscopy images of a nuclear marker enables the accurate segmentation of the cytoplasm of mammalian cells. We show that by integrating conv-nets into an image analysis pipeline, we can quantify the growth of thousands of bacterial cells and track individual mammalian nuclei with almost no manual correction. We also show that incorporation of cytoplasmic segmentation masks provides a more accurate quantification of fluorescent protein localization kinase translocation reporters (KTRs) [6]. A quantitative comparison demonstrates that conv-nets are superior to other methods, both in terms of accuracy and curation time. We show how this approach can be used to both segment and classify different mammalian cell types in a co-culture using just the phase image and a nuclear marker. We highlight particular features of our work—image normalization, segmentation refinement with active contours, and receptive field size—which were critical for conv-nets to perform robustly on live-cell imaging data using relatively small training data sets. We also explore how much recent deep learning advances—namely dropout, batch normalization, and multi-resolution networks—impact segmentation performance [37, 41, 42]. All of the software and training data described here, as well as a Docker container, is available at https://simtk.org/projects/deepcell.

### Image segmentation as image classification

**Fig 1:**
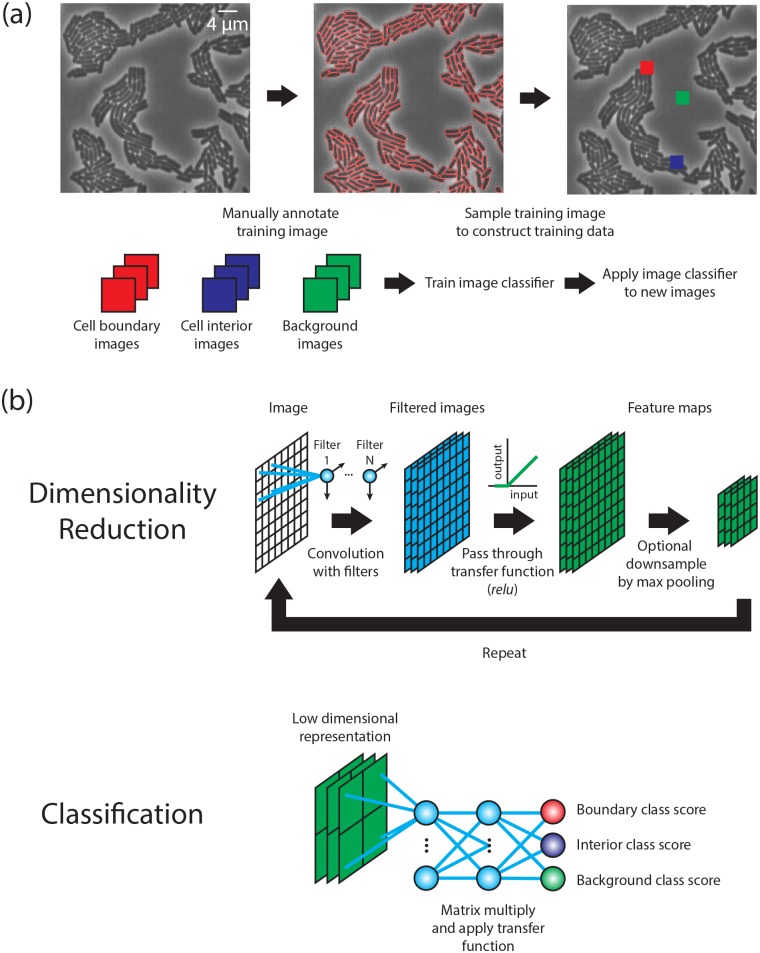
Performing image segmentation with deep convolutional neural networks. (a) Image segmentation can be recast as an image classification task that is amenable to a supervised machine learning approach. A manually annotated image is converted into a training dataset by sampling regions around boundary, interior, and background pixels. These sample images are then used to train an image classifier that can then be applied to new images. (b) The mathematical structure of a conv-net. A conv-net can be broken down into two components. The first component is dimensionality reduction through the iterative application of three operations—convolutions, a transfer function, and down sampling. The second component is a classifier that uses the representation and outputs scores for each class.

Image segmentation of single cells in microscopy images can be converted into an image classification problem [38]. Consider a manually annotated image where each pixel has been identified as either a cell boundary, cellular interior, or background (non-cell) pixel, as depicted in Fig 1a. By sampling a small region around each pixel and assigning the resulting image that pixel’s respective class, we can construct a training data set that contains representative images of each class. After the manually annotated image is reconstructed in this way, the image segmentation task is effectively reduced to finding a classifier that can distinguish between the three classes in the training data set and is robust enough to classify new images. Should such a classifier exist, any new microscope image could be segmented by decomposing it into small overlapping images, applying the classifier to each image, and then reassembling the classification prediction into a new image. This image can then be subjected to standard computer vision techniques to produce a segmentation mask—an image where the pixels for each cell’s interior have been assigned a unique integer label. Conv-nets can function as exactly such a classifier for data acquired from live-cell imaging experiments because they have both substantial representational power to encode the nonlinear relationship between images and classes, yet are general enough to provide robust predictions for new images [32, 33].

Here, we briefly review the mathematical structure of conv-nets and how they are trained to perform classification tasks [32, 38]. With respect to their mathematical structure, conv-nets can be thought of as having two components; this is schematized in Fig 1b. The first component constructs a low dimensional representation of an image using the iterative application of three operations, as depicted in Fig 1b. If we denote the input image as *I*, then the first operation is to convolve the image with a set of filters, denoted here as {*w*_1_,…, *w*_*n*_}, to produce the filtered images {*I* * *w*_1_,…, *I* * *w*_*n*_}. Each filter *w*_*i*_ has the same shape—a square with a size of 3–5 pixels—in the conv-nets considered here. The filters can be thought of as local feature extractors as their output only depends on pixels in close spatial proximity. The weights of these filters are variables—their values are changed during a training procedure to minimize the classification error on a training data set.

Once the image is filtered, the second operation is to apply a transfer function *f* to produce a set of feature maps {*f*(*I* * *w*_1_),…, *f*(*I* * *w*_*n*_)}. The transfer function serves to enable the classifier to construct non-linear decision boundaries. In this work, we use the rectified linear unit, or relu, as our transfer function which is defined as *relu*(*x*) = max(0, *x*) [43]. The third operation is an optional down-sampling using max-pooling, which creates feature maps at a coarser spatial scale. The max-pooling operation replaces an *m* × *m* window with the largest pixel value within that window. The window is then slid across the image with a particular step size to produce the down sampled image. In this work, we only consider max-pooling with a 2 ×2 window using a step size of 2. The output of these dimensionality reduction steps are a collection of down sampled (if max pooling was applied) feature maps {*pool*(*f*(*I* * *w*_1_)),…, *pool*(*f*(*I* * *w*_*n*_))}. These feature maps can be viewed as a single multi-channel image, which then serves as the input to another round of the same three operations—convolution with a set of filters, application of a transfer function, and optional down-sampling by max pooling. By iterating these three operations for several rounds (4–6 times in this work), we construct a low dimensional representation of the input image.

The second component of a conv-net, classification, uses a fully connected neural network that takes the low dimensional representation described above an input and assigns class labels. The mathematical structure is similar to a linear classifier, which collapses the low dimensional representation of the image into a single vector **r** and then multiplies it by a matrix W. The dimensions of *W* are (number of classes X number of elements in r), which by design produces the appropriate number of class scores. The output is a vector of class scores, with the highest score being called as the winner.

The difference between the linear classifier and a fully connected neural network is that the latter uses iterative applications of matrix multiplication and a transfer function to construct a nonlinear mapping between the low dimensional representation and the set of class labels. Put concretely, the first step is to collapse the low-dimensional representation into a single vector **r** and then multiply with a matrix *W*_1_. The product is then sent through the transfer function *f* to yield the output *f*(*W*_1_***r***). The repeated application of these two operations produce the output *W*_*q*_*f*(*W*_*q*-1_*f*(…*f*(*W*_1_***r***))), where the number of rows of the last matrix *W*_*q*_ is equal to the number of classes. This output is a vector of class scores which serves as the classification prediction. For the networks considered in this work, we take *q* = 3 or 4. The structure of the conv-nets explored here are shown in Table A in S1 Text.

With the mathematical structure of a conv-net defined, we now to turn to training. As with other supervised machine learning methods, conv-nets have to learn from manually annotated datasets to perform the classification task, such as the classifying of boundary, interior, and background images mentioned earlier. We refer to this dataset as our “training data” because it is used to train the conv-net as a classifier. A small portion (10%) of this dataset is set aside as “validation data” and is used to benchmark the training procedure. In the case of conv-nets, training means tuning the weights of the filters *w*_*i*_ and matrices *W*_*j*_ to minimize the classification error. This is done by first constructing a cost function that provides a reward for predicting the correct class and a penalty for predicting the wrong class for a set of images. In this work, we use soft-max cost function, which is defined as

$$Cost=-\underset{all\;images}{\sum }\text{log}\left(\frac{e^{correct\;class\;score}}{\sum_{all\;classes}e^{class\;score}}\right)+\lambda \underset{w\;\in \;\left\{all\;filter\;weights\right\}}{\sum }w^{2}.$$

The first term can be thought of as the negative log likelihood of choosing the correct class while the second is a regularization term included to reduce overfitting. With the cost function defined, batch stochastic gradient descent (or a variant like RMSprop) was used to tune the filter weights (after a random initialization) to minimize the cost function [44, 45]. Batch stochastic gradient descent works by computing the derivative of the cost function with respect to each weight for a batch of images. The cost function is minimized by adjusting the weights according to the formula

$$w=w-learning\;rate\;\times \frac{\partial Cost}{\partial w}.$$

The gradient of the cost with respect to the weights is computed analytically using the backpropagation algorithm [46]. We implemented training and execution of conv-nets in Python using the Keras, Theano, Numpy, Scipy, and Scikit-image Python packages[47–51]. Additional details about training are provided in the supplemental information.

## Results

### Conv-nets accurately segment bacterial and mammalian cells

We trained conv-nets to perform 3 different segmentation tasks—bacterial cytoplasms (from phase microscopy images), mammalian cell nuclei (from fluorescent microscopy images of cells expressing a nuclear marker), and mammalian cell cytoplasms (from phase microscopy images and images of a fluorescent nuclear marker). For the mammalian cell cytoplasm segmentation task, we opted to allow the conv-net to see both the phase image and an image of a nuclear marker. We did this for three reasons. First, most live-cell imaging experiments of mammalian cells collect images of a nuclear marker for cell tracking purposes, so we expect that data to typically be present. Second, we reasoned that because the nuclei and the cytoplasm overlap spatially, the nuclear channel might help when the phase image is ambiguous. Third, because the nuclei are typically separated spatially, we reasoned that the nuclear channel could be used to separate cells that are in close spatial proximity—either inherently by the conv-net or through a post processing step. The conv-net architectures trained on each dataset are shown in Table A in S1 Text.

Once trained, a new image can be run through the conv-net to produce a prediction of which pixels are background, cellular boundary, or cellular interior. As opposed to processing a new image patch-by-patch, d-regularly sparse kernels can be used to process images in a fully convolutional, computationally efficient fashion [35, 36]. These predictions are images with pixel values ranging from 0 to 1 to reflect how certain the conv-net is of its pixel level classification. For live-cell imaging experiments, this prediction must be turned into a binary mask—an image with a 1 at every cellular interior pixel and a 0 everywhere else. We’ve found that this necessitated an additional downstream processing step. For bacterial cells and mammalian nuclei, thresholding was sufficient to produce accurate segmentation masks. For the mammalian cytoplasm, we first used a conv-net to segment the nuclei, and then used the nuclear prediction to seed a refinement of the cellular interior prediction with active contours [52].

**Fig 2:**
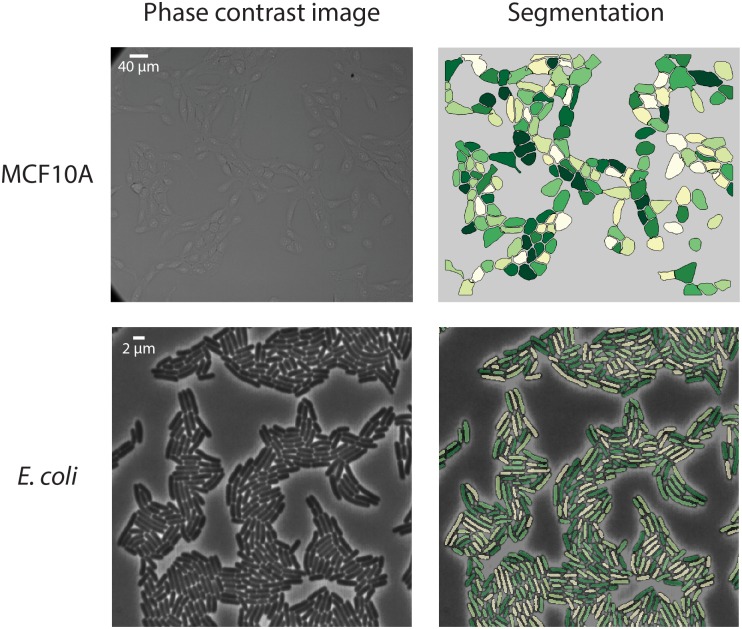
Sample images from live-cell experiments that were segmented using conv-nets. Images of bacterial and mammalian cells were segmented using trained conv-nets and additional downstream processing. Thresholding for bacterial cells and an active contour based approach for mammalian cells were used to convert the conv-net prediction into a segmentation mask.

Sample segmentation results are shown in Fig 2 and S1–S8 Figs. To date we have trained and tested conv-nets to segment images of the following cell types/lines—*E*. *coli*, NIH-3T3, MCF10A, HeLa-S3, RAW 264.7, and bone marrow derived macrophages (BMDMs). To benchmark the segmentation results, we computed standard (Jaccard and Dice) indices of segmentation accuracy [53] (Table 1). For each cell, given a reference segmentation *R* and computed segmentation *S*, the Jaccard index is defined as $JI=\;\frac{\left|R\cap S\right|}{\left|R\cup S\right|}$ and the Dice index is defined as $DI=\;\frac{2\left|R\cap S\right|}{\left|R\right|+\left|S\right|}$. The conv-net based approach has an accuracy that is either on par with or superior to the current methods developed to segment phase and fluorescent images of cellular cytoplasms and nuclei. The results in Table 1 also suggest two significant advantages of conv-nets over other approaches. First, the approach is general. Although the specifics of the architecture may need to be fine-tuned for each cell type, they have the capacity to segment images of multiple different cell lines. Second, because of their improved accuracy, less time is required to manually curate the segmentation results.

**Table 1:** Comparison of segmentation performance among different segmentation algorithms and datasets. The Jaccard (JI) and Dice (DI) indices were computed on a validation data set as previously described [53]. The Jaccard index for *E*. *coli* was estimated from the segmentation error rate (0.5% in this work and 2.6% in [17]) assuming each segmentation error was due to the incorrect joining of 2 bacteria. NA—Not available.

Cell type(s)	Method	Imaging type	JI	DI	Dense	Processing time per image	Curation time per image	Citation
**Bacteria**								
**Coliform bacteria**	Edge finding and thresholding	Phase	~0.94		Yes	5 s	~10–30 s	[15,14]
**Coliform bacteria**	Conv-nets	Phase	~0.95		Yes	120 s	1–2 s	This work
**Mammalian nuclei**								
	Thresholding and level sets	Fluorescence	0.88		Yes	2–3 s	~ 5–10 s	[45]
	Conv-nets and thresholding	Fluorescence	0.89	0.94	Yes	10 s	~0–1 s	This work
**Mammalian cytoplasm**								
**N1E115**	Voronoi diagrams	Fluorescence	0.75		No	40 s	NA	[46]
**Neural stem cells**	Level sets	Bright field	0.79		No	NA	NA	[47]
**NIH-3T3**	Edge finding, thresholding, and morphological operations	Phase		0.76–0.92	No	NA	NA	[21]
**HeLa-S3**	Ilastik (texture/edge filters and random forest classifier)	Phase	0.56*	0.71*	Yes	1s	NA	[18], This work
**HeLa-S3**	Conv-nets	DIC	0.77		Yes	1s	NA	[37]
**U373**	Conv-nets	DIC	0.93		No	1s	NA	[37]
**MCF10A**	Conv-nets and active contours	Phase	0.77	0.86	Yes	75 s	~ 2–10 s	This work
**NIH-3T3**	Conv-nets and active contours	Phase	0.77	0.86	Yes	75 s	~ 2–10 s	This work
**HeLa-S3**	Conv-nets and active contours	Phase	0.71**-0.84	0.83**-0.92	Yes	75 s	~ 2–10 s	This work

*: See the supplemental information and S17 Fig for more details.

**: For fully confluent cells. See the supplemental information and S16 Fig for more details.

**Fig 3:**
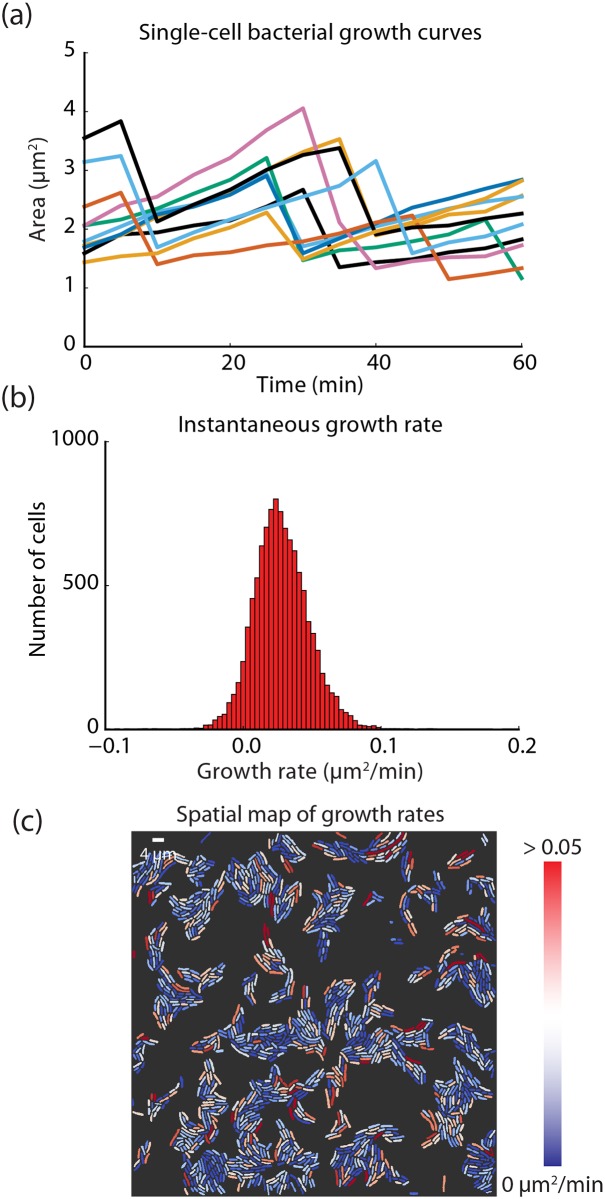
Extracting dynamic measurements of live-cell imaging experiments using conv-nets. (a) Single-cell growth curves for *E*. *coli*. Because conv-nets allow for the robust segmentation of bacterial cells, we can construct single-cell growth curves from movies of growing bacterial micro-colonies. A linear assignment problem based method was used for lineage construction. (b) By computing the change in area from frame to frame for each cell, we can construct a histogram of the instantaneous growth rate. (c) Using the instantaneous growth rate and the segmentation masks, we can construct a spatial map of growth rates with single-cell resolution. Such a map allows rapid identification of slowly dividing cells (such as metabolically inactive cells).

As a second validation test, we examined time lapse images of *E*. *coli* and HeLa cells to determine whether segmentation results were significantly different between images known to be closely related in time. This test also had the advantage of being particularly relevant to the most pressing application of our method. A linear assignment problem-based approach was used to track cells from frame to frame [54]. To estimate how many *E*. *coli* cells showed high variance in segmentation over time, we counted how many cells had a 30% or larger area increase from frame to frame (S1 and S2 Movies)—such a large increase typically means that two bacteria have been incorrectly segmented as a single cell. We found that 69 of the 11909 cells (across all frames) analyzed in Fig 3 met this criterion, which leads to an error rate of ~0.6%. This number is superior to the best results from standard approaches by four-fold (2.6%) [17]. For HeLa cells, we performed a similar analysis, but in this case comparing the segmentation contour for each cell to the actual phase image over time. We visually inspected a segmented movie (S3 and S4 Movies), and found that 21 of 145 cells tracked over time exhibited significant changes in segmentation. This leads to an error rate of 14%, which is sufficient to analyze most cells within a field of view, and represents a significant step forward in comparison to prior methods. We conclude that conv-nets are comparably accurate for a variety of cell types with a significantly reduced curation requirement.

### Conv-nets automate the quantification of live-cell imaging experiments

Having established and validated the use of conv-nets for segmentation of live-cell images, we decided to apply them to pressing needs in our field: first, constructing single-cell growth curves for bacteria; and second, quantifying localization-based kinase reporters in mammalian cells. The analysis of single-cell growth curves has recently produced a number of insights into the mechanism of bacterial growth, including the identification of the adder model for bacterial growth and the observation of metabolic co-dependence within biofilms [20, 55–58]. The images for single-cell growth curves are typically generated by growing bacteria in a patterned microfluidic device, although agar pads and other microfluidic devices have also been used [59–61]. The generation of these growth curves has typically been difficult because of the close proximity of the growing cells, although recent efforts using standard approaches have shown some success [11, 17]. By using our conv-net based approach and a linear assignment problem method for lineage construction, we can construct single-cell growth curves for *E*. *coli*–even when they are growing in a micro-colony—and use them to compute the instantaneous growth rate for each cell. These are shown in Fig 3a, 3b and S12 Fig. Because our approach can robustly produce accurate segmentation masks, it can be used to automate the analysis of thousands of bacterial cells regardless of their proximity. Using the instantaneous growth rate, we can create a spatial heat map of the micro-colony that is colored by the growth rate with single-cell resolution. This is shown in Fig 3c. We note that all of the panels in Fig 3 were generated without manual correction.

**Fig 4:**
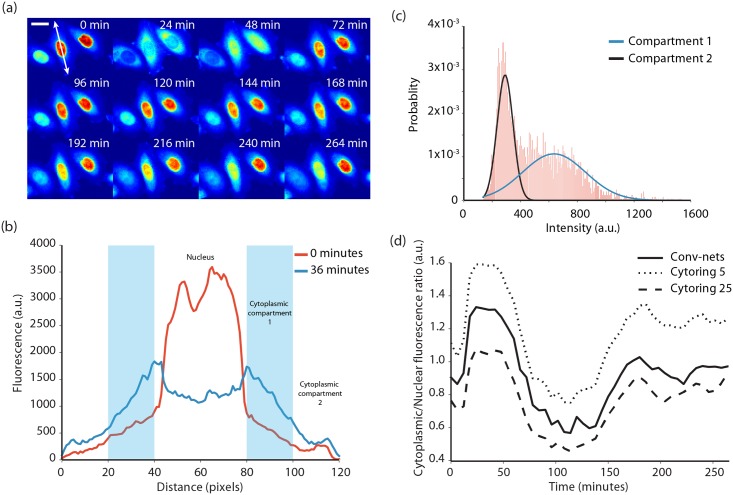
Analysis of JNK-KTR dynamics in single cells. (a) A montage of HeLa-S3 cells expressing a JNK-KTR after stimulation with TNF-α. The scale bar is 20 μm. (b) A line profile of the fluorescence of the cell highlighted in (a) which demonstrates that there is considerable spatial heterogeneity of the fluorescence in the cytoplasm. We model the cytoplasm as having two compartments, only one of which receives fluorescence from the nucleus during translocation. (c) A fit of a two component Gaussian mixture model to the cytoplasmic fluorescence of a HeLa-S3 cell. This method allows us to accurately estimate the fluorescence inside the cytoplasmic compartment that communicates with the nucleus. (d) Dynamics of the JNK-KTR after stimulation with TNF-α after segmentation with our conv-net based approach and quantification with the two component Gaussian mixture model. Plotted in comparison are dynamics obtained from using cytorings with radii of 5 pixels and 25 pixels.

A second application of our method is the quantification of localization-based kinase reporters. These reporters are a recent advance that enable the monitoring of kinase activities inside individual mammalian cells [6]. These reporters consist of a kinase recruitment domain tethered to a nuclear localization signal, nuclear export signal, and a fluorescent protein. Activation of the associated kinase leads to phosphorylation of the reporter and a nucleocytoplasmic translocation event. Because the reporter is fused to a fluorescent protein, this event results in a change in fluorescence localization. Due to the complexity of segmenting mammalian cytoplasms, we had originally taken a 5-pixel wide ring around each nucleus as a proxy for the entire cytoplasm, as has been done in other studies [27–29]. With an improved ability to sample the cytoplasm pixels, we reasoned that our conv-net based approach would lead to more accurate quantification of KTRs. To test this, we acquired movies of HeLa-S3 cells expressing the JNK-KTR after they were stimulated with TNF-α and segmented them using our conv-net approach. For this analysis, we segmented both the nuclei and the cytoplasm using conv-nets (S4 Movie). A montage of cells from this data set is shown in Fig 4a.

To identify the most robust way to quantify the nuclear-to-cytoplasm ratio, we first visually inspected our movies. This inspection revealed that the cytoplasmic fluorescence is not uniform—rather the fluorescence is highest close to the nucleus and is much lower at the periphery (Fig 4a and 4b and S3–S7 Movies). Furthermore, as evidenced by Fig 4a and 4b, the fluorescence transfer from the nucleus is spatially restricted when it enters the cytoplasm. The HeLa-S3 cytoplasm can therefore be thought of as having two compartments, only one of which receives fluorescence from the nucleus. To see how this experimental reality influences the quantitation of the data, we varied the radius of the cytoring to see how its size influences the extracted dynamics (Fig 4d and S10 Fig). We found that as we increased the radius, the qualitative shape of each curve was intact but there were important quantitative differences. These differences are important when using models to infer the concentration of active kinase inside a cell. The smallest cytoring almost exclusively samples the compartment in communication with the nucleus but also has the fewest number of pixels. The larger cytorings sample this compartment more thoroughly but also incorporate pixels that are not in the correct compartment. We therefore expect the cytoring to give qualitatively correct but quantitatively inaccurate results regardless of the radius. Our conv-net based approach improves on this method because it better samples the cytoplasmic pixels (as seen in S11 Fig). To determine the average fluorescence of the compartment that is in communication with the nucleus, we fit the pixel intensity histograms of the cytoplasm of each cell to a 2-component Gaussian mixture model as shown in Fig 4c. The resulting dynamics for this approach and comparison to the cytoring method are shown in Fig 4d. We feel this method is superior because it properly incorporates information from the entire cytoplasm whereas the cytoring forces one to make a decision between accuracy and sampling sparsity. We note that this method for quantifying KTRs may not be necessary for all cell types, as NIH-3T3 cells did not appear to have a two-compartment cytoplasm [6].

### Conv-nets enable the identification of cell type in mammalian cell co-cultures

The ability to quantify fluorescence microscopy data is a powerful application of deep learning that will lead to more accurate quantification of live-cell imaging experiments. However, conv-nets also have the ability to perform semantic segmentation—that is, to both segment individual cells and also predict their cell type [35, 62]. We explored how well conv-nets perform semantic segmentation in this context by training a conv-net on a data set that contained images of both NIH-3T3 and MCF10A cells, which exhibit distinct morphologies under phase contrast. We modified the conv-net architecture (Table A in S1 Text) by increasing the number of classes from 3 to 4 so that the conv-net could recognize the difference between the interiors of the two cell types. To generate a prediction for each cell, the trained conv-net was first applied to an image of a co-culture. We then used the sum of the NIH-3T3 and MCF10A normalized score images to generate segmentation masks as described above. We then computed the cellular classification score for each class (NIH-3T3 or MCF10A), which is given by

$$cellular\;classification\;score=\;\frac{\sum_{i\in \left\{cell\;pixels\right\}}class\;score\;for\;pixel\;i}{\sum_{classes}\sum_{i\in \left\{cell\;pixels\right\}}class\;score\;for\;pixel\;i}.$$

**Fig 5:**
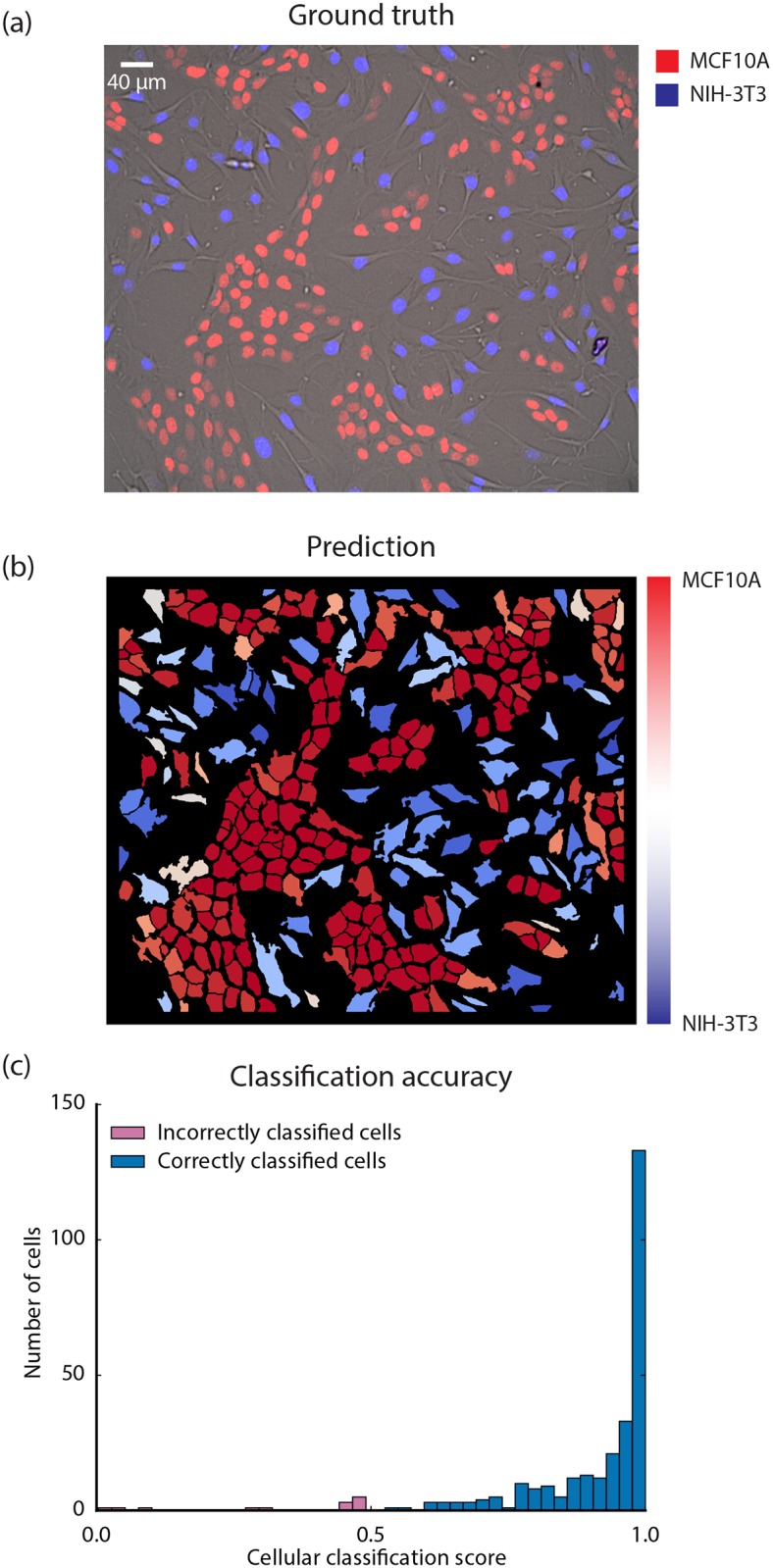
Semantic segmentation of a co-culture with MCF10A and NIH-3T3 cells. A conv-net was trained to both segment and recognize NIH-3T3 and MCF10A cells. The training data was created from separate images of NIH-3T3 and MCF10A cells with each image having a nuclear marker (Hoechst 33342) as a separate channel. (a) A ground truth image of a co-culture containing NIH-3T3 and MCF10A cells. The NIH-3T3 cells express a mCerulean nuclear marker (blue) while the MCF10A cells express an iRFP nuclear marker (red). Hoechst 33342 (image not shown) was also used to generate an image of a nuclear marker. (b) Simultaneous image segmentation and cell-type classification of the image in (a) using a trained conv-net. (c) Classification accuracy for the trained conv-net’s cellular level cell type prediction. The cellular classification score of the correct cell type for each cell is plotted as a histogram. The size of the cellular classification score is strongly associated with making a correct prediction. A classification accuracy of 86% was achieved for NIH-3T3 cells and 100% for MCF10A cells.

The results for semantic-segmentation are shown in Fig 5a and 5b. When compared with the ground truth (provided by distinct nuclear markers for the two cell types), our trained conv-net has an accuracy of 95% when it comes to a cellular level prediction of cell type. One image containing 92 NIH-3T3 cells and 182 MCF10A cells was classified to determine the accuracy of cell type classification. Similar results were obtained for additional images (see S9 Fig).

We were curious whether stronger cellular classification scores were less prone to classification errors. To explore this, we constructed a histogram of the cellular classification scores of the correct class for each cell (Fig 5c). We found that classification errors are clustered around 0.5, with only 3 cells with errors having a value less than 0.25. This observation leads us to conclude that the closer a cellular classification score is to 1, the more likely that the prediction is correct.

## Discussion

### Advantages of conv-nets over standard computer vision techniques

Image segmentation is the core task required to convert the data from live-cell imaging experiments into a quantitative, dynamic description of living systems with single-cell resolution. Here, we have shown that deep learning can perform this task with accuracy sufficient for live-cell experiments. The approach to single-cell segmentation we have outlined has four main advantages. First, incorporating conv-nets into an image segmentation pipeline significantly reduces the time required for manual supervision and correction. This improvement comes about from the improved accuracy of the segmentation masks. In the two case studies presented here, we were able to obtain our end result with no manual curation. We note that this ability depends on data quality and likely will not hold for all experiments. To get a sense of the actual time savings, we estimated the curation time required for our previous work and searched the literature to obtain estimates of how long it takes to analyze a typical experiment [4, 5, 9]. We found that, including parameter tuning, for nuclear segmentation we typically spend 6–12 hours analyzing a typical data set (96 positions x 45 frames) using a watershed based segmentation approach. Analyzing the segmentation results for bacterial cell division to produce the results in [15] required approximately 40 hours. Similarly, manually segmenting the cytoplasms of 3T3 cells in ~200 images required over 200 hours [26]. Even in the cases where the computational cost is significant, conv-nets invariably lead to significant savings in human time. Moreover, human time and computation time are not equivalent—a 20 hour task may take a human nearly a week (given scheduling demands and ergonomic requirements) while a computer can complete it within a day (or less if parallelization is used).

The second advantage is the low time and hardware requirements for training and testing new conv-nets. We have found that for the training data we generate, it typically takes 2–4 hours to generate training data (human time) and 5–10 hours (computer time) to train the conv-net architectures considered here. Both of these represent a fixed initial cost which apply to all subsequent experiments for a given cell type or types. In addition, because a capable GPU is the key piece of hardware required for this work, it is relatively inexpensive (~500–1000 US dollars) to provide existing workstations with the capacity to train and execute conv-nets.

Third is the ability to perform semantic segmentation to identify different cell types without sacrificing an additional fluorescence channel. This ability enables the analysis of live-cell imaging experiments involving co-cultures, monitoring differentiation, or possibly even tissues.

Fourth, and most importantly, is the generality of the approach. Using our framework, segmenting new cell types involves simply creating a new training data set and training a new conv-net. To date we have been able to segment images from 5 different mammalian cell lines with no changes in the conv-net architecture. While the architecture may need refinement, deep learning will likely be applied to even more cell types and enable the analysis of a variety of live-cell imaging experiments.

### Design rules for new conv-nets

Moving forward, we foresee two possible approaches. In one approach, similar to the ImageNet challenge, one large network would be trained to segment and classify the majority of cell lines that are in use today [33, 35]. In another approach, which we consider to be more likely, a collection of conv-nets would be designed for each specific segmentation task required for a live-cell imaging experiment. This approach we term laboratory scale deep learning. By making the trained conv-nets and the requisite training data publically available, laboratories performing this type of work would be able to build on each other’s technical advances. In pursuit of this aim, here we outline some of the design rules we’ve extracted from our successes (and failures) in training conv-nets.

First, we found that image normalization was critically important for robustness, segmentation performance, and processing speed. We tried three different normalization schemes—normalizing images by the median pixel value, by the maximum pixel value, or not at all. We found that normalizing by the median pixel value was best with respect to robust performance on images acquired with different illumination intensities. In the case of semantic segmentation, we found that without proper normalization, the conv-nets learned intensity differences between the MCF10A and NIH-3T3 data sets instead of differences in cell morphology. This led to poor performance when the conv-nets were applied to new images, as they classified all the cells as the same cell-type based on the images illumination (see S13 Fig). Furthermore, the normalization choice impacts whether or not a fully convolutional implementation of conv-nets can be used when segmenting new images. Normalizing patches by their standard deviation, for instance, or performing PCA based whitening would require new images to be processed in a patch-by-patch manner. We note that in our experience, performance was optimal when the illumination intensity was reasonably similar to or higher than the illumination used to acquire the training data.

Second is the importance of data augmentation. One of our key results is that only a few hundred cells worth of training data is required to produce a well performing conv-net. This is in contrast to the thousands of images contained in the ImageNet and PASCAL databases. Smaller training datasets were sufficient for out purposes because we made maximal use of the data available by using data augmentation through image rotation and reflection and by enabling the conv-net to “see” the nuclear channel—information that is collected in every live-cell imaging experiment done in our lab. Curiously, we found that using image shearing for data augmentation did not significantly improve the segmentation performance (Table B in S1 Text). While additional data augmentation techniques may improve performance, it is clear that the large numbers of images typically used to train conv-nets are not required for this purpose. Nevertheless, we anticipate that the incorporation of additional training data will lead to more accurate and robust conv-nets in the future.

Third, is the importance of hyper-parameters. The most important hyper-parameter in our experience was the conv-net’s receptive field size, or the size of the image patches sampled from the training image. We found that this was a key hyper-parameter that had to be tuned to obtain a well-performing conv-net. Our experience leads us to believe that the optimal size is roughly on the order of the diameter of a cell. Receptive fields that are too small led to conv-nets that classified the background as edges while receptive fields that were too large took longer to train and are more prone to over-fitting. While we obtained good results given our choice of hyper-parameters (for instance the regularization strength—see S15 Fig), we note that optimization of the number of layers in a conv-net, the number of filters in each layer, the filter sizes for each layer, or the conv-net architecture may also lead to further improvements [35, 37, 63].

Fourth, we found that segmentation refinement was necessary to produce segmentation masks that could be applied to real data. While conv-nets produce remarkably accurate pixel-wise classification predictions, we found we still needed to refine these predictions to produce binary segmentation masks. In the end, the method we found that worked the best was using the conv-net predictions to guide active contours. One advantage of this novel approach was that we could seed this process using the segmentation masks of the cell nuclei, in essence using the cell nuclei to separate the masks of spatially proximate cells. While it is possible that additional training data and hyper-parameter optimization may make this refinement step unnecessary, this is likely an area for further improvement. For instance, conditional random fields have shown success in refining conv-net predictions for semantic segmentation and may prove to be of some use here [62]. Incorporating segmentation and tracking together in a unified framework may also provide opportunities for performance improvement [17].

Fifth, we explored how much dropout, batch normalization, and multi-resolution fully connected layers, improve the segmentation performance of conv-nets. Dropout is a regularization technique in which filters are randomly turned off during training [41]. By training in this fashion, the conv-net learns to not depend too heavily on any one filter. Batch normalization is a solution to the covariate shift problem that works by using per batch statistics to convert the output of each conv-net layer into one that has zero mean and unit variance [42]. Batch normalization also functions as a regularization method. Multi-resolution fully connected layers are an advance geared towards improving semantic segmentation performance [34, 35, 37]. By allowing hidden layers to see multiple spatial scales, these networks can boost their segmentation performance. We added these features to conv-nets while keeping most of their features fixed, trained them on our HeLa-S3 training data, and then evaluated their performance (in conjunction with active contours) on a validation data set. The classification error during training (on both training and validation data) is shown in S14 Fig and the results are shown in Table B in S1 Text. We found that performance for most of these variations were similar. Curiously, conv-nets with dropout and multi-resolution fully connected layers had worse performance than an unmodified conv-net. Batch normalization in conjunction with multi-resolution layers produced the best performance, but these conv-nets were prone to overfitting (as judged by the difference between the training and validation classification error). The networks presented here use batch-normalization, which we opted for because of their regularization ability, their reduced training time, and a desire to avoid overfitting.

### Conclusions

Deep learning has been a transformative technology for computer vision, and based on our experiences outlined in this paper, we expect that it will be a powerful tool for analyzing images from live-cell imaging experiments. We offer several thoughts in conclusion. First, we expect that laboratory scale deep learning (as described above) will the development model moving forward. Second, we expect that the availability of open source deep learning APIs, such as Keras (as used in this work), Theano, Torch, Caffe, and TensorFlow, in addition to the myriad of tutorials available online, will help make this technology more accessible to the causal user. Finally, we expect that in addition to being able to better quantify fluorescence levels, there are other classes of live-cell imaging experiments where the localization of fluorescence must be quantified that will benefit from the approach outlined here [27, 64–66]. Given our experience detailed in this work, we expect deep learning to have a transformative impact on the analysis of live-cell imaging experiments.

## Materials and Methods

### Constructing training datasets

To construct manually annotated datasets for *E*. *coli* strain MG1655 and 5 different mammalian cell lines (NIH-3T3, MCF10A, HeLa-S3, RAW 264.7, and bone marrow derived macrophages), we first acquired images to construct training datasets. For bacterial cells we acquired phase images of the bacterial micro-colonies while for mammalian cells, we acquired both fluorescent images of labeled nuclei and phase images of the cytoplasm. We then used ImageJ to annotate each training dataset and classify each pixel in a training image as either boundary, cellular interior, or background. Details of this annotation procedure are contained in the supplemental information. Empirically, we found that annotating one microscope image containing a total of ~300 bacteria, ~500 nuclei, or ~ 100 mammalian cells was sufficient to make a training dataset that led to a well performing conv-net; the annotation typically required ~2–4 hours. A small patch (31x31 pixels for bacteria, and 61x61 pixels for mammalian cytoplasm and nuclei) was sampled around each annotated pixel to create a collection of representative images for each class. We found that this sampling was best done at run time to preserve GPU memory. This collection of images was then randomly down-sampled so that each class had equal representation in the collection.

One concern regarding the performance of trained conv-nets we had was their robustness to fluctuations in illumination and microscope cameras. Sharable solutions to this segmentation problem require some amount of robustness to these changes, as different labs use different microscopes and cameras to acquire data. Even within labs, image acquisition parameters may not be identical between experiments. To account for this, we normalized our training data sets prior to using them to train a conv-net. Each training image was first divided by the median pixel value. The local average of this normalized image was computed by applying an averaging filter (the same size as that of the neural network’s effective receptive field size—in our case 31x31 for bacterial cells and 61x61 for mammalian nuclei and cytoplasms) and then subtracted from the normalized image to create the final image that was sampled to train a conv-net. After sampling our image, we then performed data augmentation by randomly rotating each patch by either 0, 90, 180, or 270 degrees and randomly reflecting them on the fly prior to their use our training dataset. Our reasoning was that class labels should be invariant to these transformations.

After normalization and data augmentation, each training dataset consisted of ~200,000–400,000 patches. The mammalian cytoplasm training datasets contained two channels—a phase image and an image in which cell nuclei were labelled with Hoechst 33342 (NIH-3T3 and MCF10A) or H2B-iRFP (HeLa-S3).

### Segmenting new images

To construct a segmentation mask for a new image, the trained conv-net is applied to the entire image to produce pixel level classification predictions. This is done by using a fully convolutional implementation of conv-nets that can be directly applied to the entire image [35, 36]. We also used model parallelism (averaging the results of 5 trained networks) to improve our segmentation accuracy.

While the classification predictions produced by conv-nets were remarkably accurate, we found that they required further refinement to produce binary segmentation masks. As opposed to using the raw class scores for each pixel as the input for refinement, we instead use the softmax normalized score $\frac{e^{class\;score}}{\sum_{all\;classes}e^{class\;score}}$, which we loosely interpret as the probability deduced by the conv-net that the pixel in question belongs to that particular class. For bacterial cells, we found that thresholding of the softmax normalized score for the cell interior class with a value of 0.6 was sufficient to produce accurate masks. For nuclei, we found that thresholding with a value of 0.5–0.75 in a similar fashion to bacteria worked well in most cases, with adaptive thresholding of the softmax normalized cell interior score being used for images with very dim nuclei. For mammalian cells, the conv-net prediction in combination with the nuclear marker was used to guide an active contour algorithm to further refine the segmentation[52]. Training error and segmentation results for *E*. *coli*, MCF10A, NIH-3T3, and HeLa-S3 cells are shown in Fig 2, S1–S8 Figs and S4 and S7 Movies.

## Supporting Information

S1 Text Supplemental text. (DOCX)

S1 Fig Sample image, conv-net interior prediction map, segmentation mask, and training/validation error curves for *E*. *coli*. (TIF)

S2 Fig Sample image, conv-net interior prediction map, segmentation mask, and training/validation error curves for MCF10A cells. (TIF)

S3 Fig Sample image, conv-net interior prediction map, segmentation mask, and training/validation error curves for NIH-3T3 cells. (TIF)

S4 Fig Sample image, conv-net interior prediction map, segmentation mask, and training/validation error curves for HeLa-S3 cells. (TIF)

S5 Fig Sample image, conv-net interior prediction map, segmentation mask, and training/validation error curves for RAW 264.7 cells. (TIF)

S6 Fig Sample image, conv-net interior prediction map, segmentation mask, and training/validation error curves for bone marrow derived macrophages. (TIF)

S7 Fig Sample image, conv-net interior prediction map, segmentation mask, and training/validation error curves for H2B-iRFP labeled and DAPI stained nuclei. (TIF)

S8 Fig Sample image, conv-net interior prediction map, segmentation mask, and training/validation error curves for semantic segmentation of MCF10A cells and NIH-3T3 cells. (TIF)

S9 Fig Additional semantic segmentation of NIH-3T3 and MCF10A cells. 286 cells were analyzed, including 93 3T3 cells and 192 MCF 10A cells. The classification accuracy was 89% for NIH-3T3 cells and 98% for MCF10A cells.
(TIF)

S10 Fig Sensitivity analysis of the influence of the cytoring size on the dynamics of the JNK-KTR. While the qualitative shapes of the curves remain intact as the size of the cytoring increases, there are important quantitative differences that emerge as the cytoring increases. In the grey trace, the first peak is identified as being quantitatively identical to the other cells in the plot if a 5 pixel cytoring is used, but becomes separated from the rest as the cytoring size is increased. A similar quantitative change can be seen in the size of the second peak in the purple trace, as it quantitatively becomes more diminished as the cytoring size is increased.
(TIF)

S11 Fig Areas of the different cytorings used in S10 Fig. (TIF)

S12 Fig Histogram of the instantaneous growth rate for a bacterial micro-colony. This histogram is identical to the histogram showed in Fig 3, with the axes expanded to show the negative growth rates corresponding to cell division.
(TIF)

S13 Fig Poorly performing conv-nets. This figure highlights the importance of image normalization and receptive field size in training robust conv-nets. (a), (b), and (c). Mammalian-net semantic was trained on an un-normalized images of NIH-3T3 and MCF10A cells. Instead of learning differences in cell shape, the conv-net learned the brightness difference between the two dataset sources, leading to poor performance on images of co-cultures. 100% of MCF10A cells and 14% of 3T3 cells were classified correctly. (d) A conv-net with a 41x41 receptive field (as opposed to the 61x61 receptive field of mammalian net) was trained using a HeLa cell data set. Because of the smaller receptive field, the conv-net has difficulty distinguishing cell boundaries and interiors from background.
(TIF)

S14 Fig Training and validation error for conv-nets to assess the performance improvements provided by dropout, batch normalization, shearing for data augmentation, and multi-resolution fully connected layers. Dropout was only used in fully connected layers. The segmentation performance of each network as quantified by the Jaccard and Dice indices is provided in Table B in S1 Text.
(TIF)

S15 Fig Regularization optimization. The L2 regularization parameter was varied from 0, 10^−7^, 10^−6^, and 10^−5^. Lower regularization was associated with more fluctuations in the classification error on the validation data set.
(TIF)

S16 Fig Segmentation accuracy vs. cell density for HeLa-S3 cells. 1250, 2500, 5000, 10000, and 20000 cells were plated in the wells of a 96 well dish and imaged. The images were segmented manually and with conv-nets to compute the Jaccard and Dice indices. Segmentation performance remains stable for most plating densities, but decreases once cells are confluent.
(TIF)

S17 Fig Comparison of segmentation performance of conv-nets and Ilastik on a HeLa cell validation data set. (a) Phase image of HeLa cells. (b) Ground truth for the cell boundary. (c) Conv-net soft-max score for edge and interior prediction. (d) Ilastik segmentation. (e) Conv-net segmentation after active contour processing. (f) Ilastik segmentation after active contour processing.
(TIF)

S1 Movie Phase images of a growing *E*. *coli* micro colony. (AVI)

S2 Movie Bacteria-net output when used to process S1 Movie. (AVI)

S3 Movie Phase images of HeLa-S3 cells expressing the JNK-KTR. (AVI)

S4 Movie Segmentation of S3 Movie. (AVI)

S5 Movie Nuclear marker channel for S3 Movie. (AVI)

S6 Movie JNK-KTR channel for S3 Movie. (AVI)

S7 Movie Representative movie of HeLa-S3 with overlaying nuclear marker and segmentation boundaries. (AVI)
