# Supplementary material for: Deep Learning Automates the Quantitative Analysis of Individual Cells in Live-Cell Imaging Experiments
Source: PLoS Comput Biol. 2016 Nov 4;12(11):e1005177. doi: 10.1371/journal.pcbi.1005177 (PMC5096676; doi:10.1371/journal.pcbi.1005177)
Supplement: S1 Text — (DOCX) [file pcbi.1005177.s001.docx]

**Supplementary information**

**Cell line acquisition**

We used *E. coli* strain MG1655 and mammalian cell lines NIH-3T3, HeLa-S3, and MCF10A for this work. The *E. coli* strain was acquired from ATCC. The NIH-3T3 cell line was acquired from ATCC. The HeLa-S3 cell line was acquired from the Wysocka lab at Stanford and was originally purchased from ATCC. The MCF10A cell line was acquired from the Meyer lab at Stanford. The cells have not been authenticated and were not tested for mycoplasma contamination.

**Image acquisition**

*E. coli* strain MG1655 was inoculated from a -80^0^C culture and grown overnight in LB media. The culture was then inoculated into 5 mL of M9sup (1X M9 salts, 1 mM thiamine hydrochloride, 0.4% glycerol, 0.2% casamino acids, 2mM MgSO_4_, 0.1 mM CaCl_2_) and grown for ~3 hours to an OD600 of 0.2. The cells were then pelleted by centrifugation at 3000g’s for 5 minutes and re-suspended in 500 uL of M9sup. Agar pads (M9sup + 1.5% agarose) were prepared as previously described. 1uL of cells was spotted onto the agar pad and allowed to dry for 5 minutes. The agar pad was then inverted, placed inside a LabTek slide, and then imaged on a Nikon Ti-E inverted microscope equipped with environmental control (37 °C), a 100X phase objective, and an Andor Neo 5.5 CMOS camera using 2x2 binning. Images were taken every 5 minutes with a 20 ms exposure time.

Mammalian cells were cultured in Dulbecco’s modified Eagle’s medium (DMEM, Invitrogen) supplemented with 2mM L-Glutamine (Gibco), 100 U/ml penicillin, 100μg/ml streptomycin (Gibco), and either 10% fetal bovine serum (Omega Scientific) for HeLa-S3 cells, or 10% calf serum (Colorado Serum Company) for NIH-3T3 cells. MCF-10A cells were cultured in DMEM/F12 with L-Glutamine (Invitrogen) supplemented with horse serum (Invitrogen, 5%), EGF (Peprotech, 20ng/ml), hydrocortisone (Sigma, 0.5μg/ml), cholera toxin (Sigma, 100ng/ml), insulin (Sigma, 10μg/ml), (Gibco), 100 U/ml penicillin, 100μg/ml streptomycin (Gibco).

For imaging, mammalian cells were seeded onto fibronectin (Sigma, 10ug/ml) coated glass bottom 96-well plates (Nunc) and allowed to attach overnight. Media was removed and replaced with imaging media (FluoroBrite DMEM (Invitrogen) supplemented with 10mM Hepes, 1% FBS, 2mM L-Glutamine) at least 1 hour prior to imaging. Cells were then incubated with imaging media containing WGA Alexa-Fluor 594 (Invitrogen, 4μg/ml) for 10 minutes, rinsed twice and fresh imaging media was then added. Cells without a genetically encoded nuclear marker were incubated with 50ng/ml Hoechst (Sigma) prior to imaging. Cells were imaged with a Nikon Ti-E fluorescence microscope with environmental control (37°C, 5% CO_2_) and controlled by Micro-Manager. Images were acquired with a 20x objective (40x for RAW 264.7 cells) and an Andor Neo 5.5 CMOS camera with 2x2 binning, and with a z-section acquired at -10μM for the phase image. For time-lapse experiments HeLa-S3 cells containing H2B-iRFP670 and JNK-KTR-Clover were stimulated with TNF-α (Roche, 10ng/ml) and images were acquired at 6 minute intervals.

**Constructing training datasets**

Training datasets were constructed by manually segmenting images using ImageJ. We used either a mouse or a Wacom Intuos Draw graphics tablet (Model #CTL490DW ~ $100) to perform the segmentation. Cell boundaries were traced using the freehand tool. ImageJ’s ROI manager feature was used so that a single image could be manually segmented in a single session. After the contours were traced, we then created a new image in ImageJ and used the “draw” command to create an image of the cell boundaries. The cell boundary image was then saved. We then used the “flood fill” tool to fill in the boundaries and create a preliminary mask of the cell interior. We then opened the saved image of the cell boundaries and used the “image calculator” command to subtract the boundaries from the preliminary mask of the cell interior to create the final mask. The final mask was then saved. We found that a trained technician required ~2 hours to segment 1 field of view of mammalian cells (~100 cells) and ~4 hours to segment 1 field of view of bacterial cells (~400 cells). The raw phase image, nuclear marker image (for mammalian cells), cell boundary image, and cell interior image constituted the input to create each training data set.

Once the training images were manually annotated, they were then processed to create training data. The training data for all the different cell types was constructed in a similar fashion. First, the cell boundary masks were enhanced using a morphological dilation operation with a disk structuring element 1 pixel in diameter. The cell interior mask was subtracted from the cell boundary mask to ensure that each pixel in the training data had a unique classification. Each channel in the training image was then normalized by first dividing by the 50^th^ percentile pixel value and then subtracting an image of a local mean that was obtained by applying an averaging filter. The dimensions of the averaging filter were the same as the conv-net’s receptive field – i.e. 31 x 31 for bacterial images and 61 x 61 for everything else. This modification was chosen because executing a conv-net in a fully convolutional manner requires that the normalization method take the entire image as an input, not small windows. The cell interior, cell boundary, and background images were then used to obtain representative images for each class. Because we desired to have equal representation among classes, we subsampled the interior and background image collection so that they contained the same number of images as the boundary collection. Rather than storing each sampled image, we instead stored the pixel locations and then sampled the training data image and performed data augmentation (image rotation and reflection) on the fly. We found this significantly reduced the amount of GPU memory necessary. ~10% of the images were set aside and used for validation during training. Images and the pixel locations were saved as a Numpy array in the npz format.

**Upstream and downstream processing**

New images were normalized in the same fashion as the training data (dividing by the 50^th^ percentile pixel value and subtracting an image of the local mean) and then processed using a fully convolutional implementation of the trained conv-nets (which allowed for a 2 order of magnitude speed up in the run time). For each image we process, we process it with 5 trained conv-nets and average the resulting normalized softmax scores. We found that using model parallelism in this fashion led to more robust segmentation masks.

Generating pixel level classification predictions using conv-nets is computationally expensive if the input image is divided into windows, processed with a trained conv-net, and the predictions reassembled into an image. The high computational cost is due to neighboring windows having a high degree of overlap (~95% identical pixels in the worst case), which leads to redundant computations. This computational cost becomes prohibitive as 1280 x 1080 images require over 30 minutes for processing on a GTX 980. To process net images with trained conv-nets, we use convolutions and pooling with d-regularly sparse kernels as has been described in prior works [35, 36]. These kernels allow for a fully convolutional implementation that can process an entire image at once, as opposed to processing each window separately.

The interior softmax normalized score was then used as the basis for an additional segmentation step – thresholding for bacterial and nuclear images and active contours for mammalian cytoplasm images [52]. Conv-nets used to identify individual nuclei in the nuclear marker image – this image was used as markers to seed the active contour segmentation. Because a fully convolutional implementation was used, 1280 x 1080 images typically processed by the conv-nets in ~75 seconds on an NVidia GTX980 graphics card. Fluorescence images were flat fielded and had the background fluorescence subtracted prior to quantification. A linear assignment problem based approach was used to link cells from frame to frame [54].

**Semantic segmentation upstream and downstream processing**

For semantic segmentation, the sum of the 3T3 and MCF10A softmax normalized score images were used as the interior image. This image was then processed the same manner as described above. For each cell, its cell type score was computed by the formula

$$cell type score i= \frac{\sum_{j \in\{all pixels\}} cell type score i for pixel j}{\sum_{k \in\{all cell types\}} \sum_{j \in\{all pixels\}} cell type score k for pixel j}.$$

**Details of training conv-nets**

Our software to train and execute conv-nets was developed in Python 2.7 using the Keras and Theano packages. For convolutional neural networks without batch normalization, we used the RMSprop optimization algorithm for training [44]. Briefly, RMSprop utilizes the magnitudes of previously calculated gradients to normalize the gradient computed during each training step. During training, for each parameter a cache of gradient magnitudes is kept and updated each step according to the rule

$$cache_{i}\to decay\_rate\times cache_{i}+\left( 1-decay\_rate \right)\times\left( \frac{\partial cost}{\partial w_{i}} \right)^{2}.$$

The cache is initialized with each parameter’s initial value and then updated with the above rule. Each weight $w_{i}$ is then updated according to the rule

$$w_{i}\to w_{i}-\frac{learning\_rate}{\sqrt{cache_{i}+ \varepsilon}}\times\frac{\partial cost}{\partial w_{i}}$$

We also used learning rate annealing to decrease the learning rate during training. Every epoch, the learning rate was updated according to the rule

$$learning\_rate\to learning\_decay\_rate\times learning\_rate.$$

During training, we used a batch size of 256 images, a decay rate of 0.95, an $\varepsilon$ of 10^-8^, a learning rate of 0.001, a learning decay rate of 0.95-0.98, and a regularization parameter of 10^-5^. Networks were trained for 25 epochs, where one epoch is one iteration through the entire training data set.

For networks with batch normalization, we used the standard stochastic gradient descent algorithm outlined in the main text with momentum­. We used a batch size of 256 images, a learning rate of 0.01, momentum of 0.9, learning rate decay of 0.95 (per epoch), and a regularization parameter of 10^-5^. Networks were trained for 25 epochs. We found that batch normalized networks did not train well with smaller batch sizes.

**Benchmarking**
To benchmark segmentation performance, we used the Jaccard and Dice indices as described in the main text. To estimate the Jaccard index for bacterial cells, we first estimated the segmentation error rate – 0.6% for conv-nets in this work and 2.6% (8 mis-segmented cells identified in a field of 310 cells) for the method described in [17]. Because the failure mode is usually identifying two neighboring cells as one cell, the Jaccard index can be approximated as

$JI=\left( 1-err \right)*0.95+err*0.5$.

To benchmark Ilastik, we manually segmented one field of view of HeLa cells using the Ilastik interface and used it to train the Ilastik pixel classifier. The trained classifier was then run on our validation data set for HeLa cells – the results are shown in Figure S17. Because the result was not of sufficient quality to produce a segmentation mask by thresholding, we then subjected it to the same downstream processing as the conv-net (active contours seeded by the cell nuclei). The resulting segmentation mask was used to compute the Jaccard and Dice indices. To estimate the manual curation time, we polled users in the Covert lab how long it took them to identify and (optionally) correct segmentation errors that arise from the different methods. Curating segmentations of mammalian nuclei produced by a thresholding/watershed algorithm required about 5-10 s per image. Curating (with correction) segmentations of bacterial cells growing in a mother machine required 42 man hours of curation for 5000 frame videos of 300 cells [15]. We estimate that this translates into ~10-30 s of curation per frame of an equivalent movie that has 300 cells in each frame.

**Source code**

All source code, manually annotated training data, and manually annotated validation data is available on our lab’s Simtk repository at http://simtk.org/projects/deepcell.

**Supplementary tables**

Table A: Topologies for the neural network architectures used in this paper.

| Feature net 31x31 (bacteria) | | Feature net 61x61 | |
| --- | --- | --- | --- |
| Layer | **Filter dimensions** | **Layer** | **Filter dimensions** |
| Layer 1 – Convolution | 32 x 1 x 4 x 4 | **Layer 1 – Convolution** | 64 x 2 x 3 x 3 |
| Layer 2 – Batch normalization |  | **Layer 2 – Batch normalization** |  |
| Layer 3 – Activation (ReLu) |  | **Layer 3 – Activation (ReLu)** |  |
| Layer 4 – Max Pooling | 2x2 | **Layer 4 - Convolution** | 64 x 64 x 4 x 4 |
| Layer 5 - Convolution | 64 x 32 x 3 x 3 | **Layer 5 – Batch normalization** |  |
| Layer 6 – Batch normalization |  | **Layer 6 – Activation (ReLu)** |  |
| Layer 7 – Activation (ReLu) |  | **Layer 7 – Max Pooling** | 2x2 |
| Layer 8 - Convolution | 64 x 64 x 3 x 3 | **Layer 8 - Convolution** | 64 x 64 x 3 x 3 |
| Layer 9 – Batch normalization |  | **Layer 9 – Batch normalization** |  |
| Layer 10 – Activation (ReLu) |  | **Layer 10 – Activation (ReLu)** |  |
| Layer 11 – Max Pooling | 2x2 | **Layer 11 – Convolution** | 64 x 64 x 3 x 3 |
| Layer 12 - Convolution | 128 x 64 x 3 x 3 | **Layer 12 – Batch normalization** |  |
| Layer 13 – Batch normalization |  | **Layer 13 – Activation (ReLu)** |  |
| Layer 14 – Activation (ReLu) |  | **Layer 14 – Max Pooling** | 2x2 |
| Layer 15 – Convolution | 200 x 128 x 3 x 3 | **Layer 15 - Convolution** | 64 x 64 x 3 x 3 |
| Layer 16 – Batch normalization |  | **Layer 16 – Batch normalization** |  |
| Layer 17 – Activation (ReLu) |  | **Layer 17 – Activation (ReLu)** |  |
| Layer 18 – Fully connected | 200 x 200 | **Layer 18 – Convolution** | 64 x 64 x 3 x 3 |
| Layer 19 – Batch normalization |  | **Layer 19 – Batch normalization** |  |
| Layer 20 – Activation (ReLu) |  | **Layer 20 – Max Pooling** | 2x2 |
| Layer 21 – Fully connected (compute class scores) | 3 x 200 | **Layer 21 – Convolution** | 200 x 64 x 4 x 4 |
| Layer 22 – Activation (softmax) |  | **Layer 22 – Batch normalization** |  |
|  |  | **Layer 23 – Activation (ReLu)** |  |
|  |  | **Layer 24 – Fully connected** | 200 x 200 |
|  |  | **Layer 25 – Batch normalization** |  |
|  |  | **Layer 26 – Activation (ReLu)** |  |
|  |  | **Layer 27 – Fully connected (compute class scores)** | 3 (or 4) x 200 |
|  |  | **Layer 28 – Activation (softmax)** |  |

Table B: Effect of dropout, batch-normalization, multi-resolution hidden layers, and shearing on segmentation performance.

| Feature | Dice index | Jaccard index |
| --- | --- | --- |
| None | 0.847 | 0.917 |
| Dropout | 0.838 | 0.912 |
| Batch normalization | 0.845 | 0.916 |
| Multi-resolution hidden layers | 0.835 | 0.910 |
| Batch normalization + multi-resolution hidden layers | 0.853 | 0.920 |
| Shearing | 0.848 | 0.918 |
