# Supplementary figures and images for: Deep Learning Automates the Quantitative Analysis of Individual Cells in Live-Cell Imaging Experiments
Source: PLoS Comput Biol. 2016 Nov 4;12(11):e1005177. doi: 10.1371/journal.pcbi.1005177 (PMC5096676; doi:10.1371/journal.pcbi.1005177)

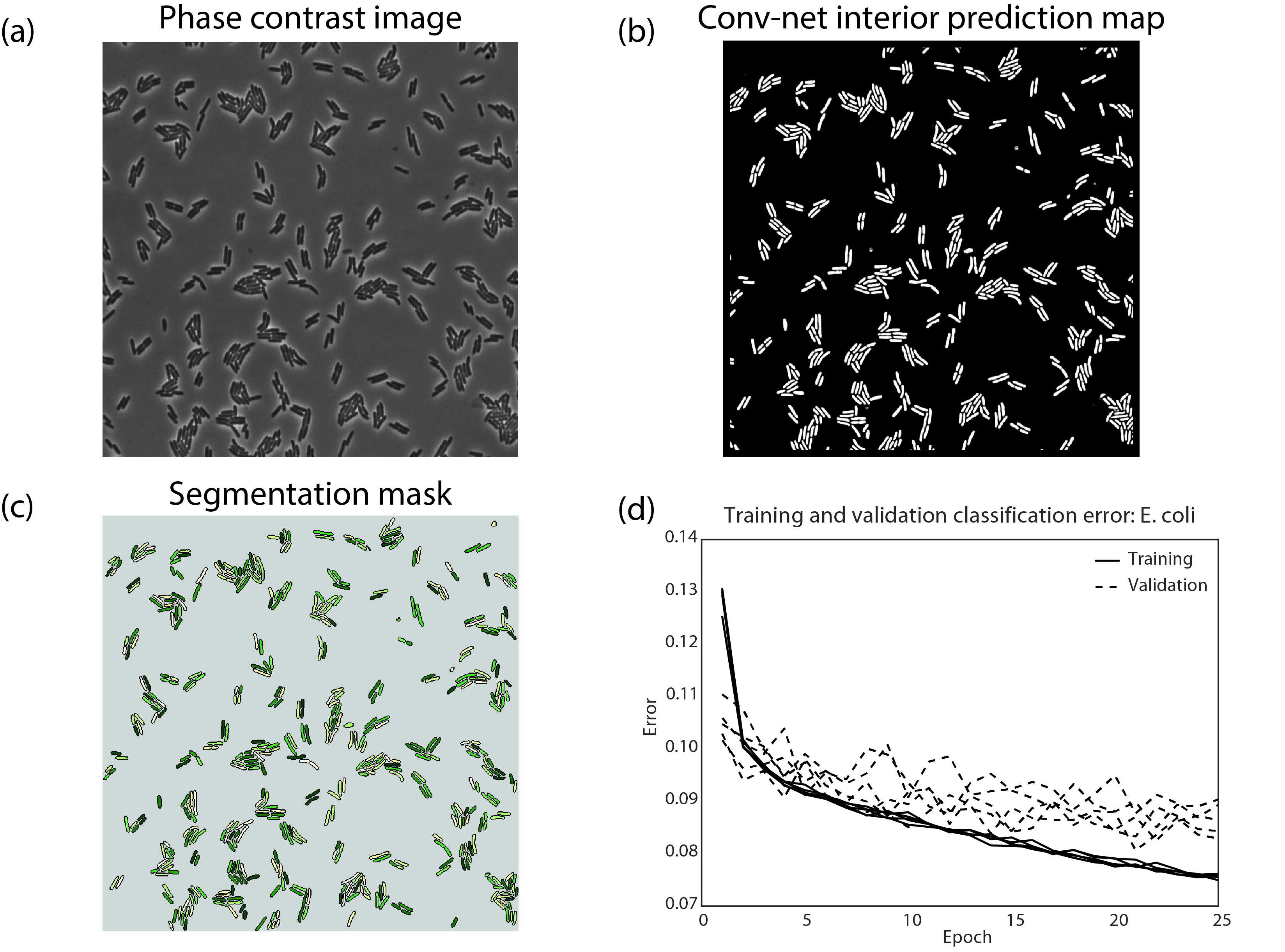

Supplement: S1 Fig — (TIF) [file pcbi.1005177.s002.tif]

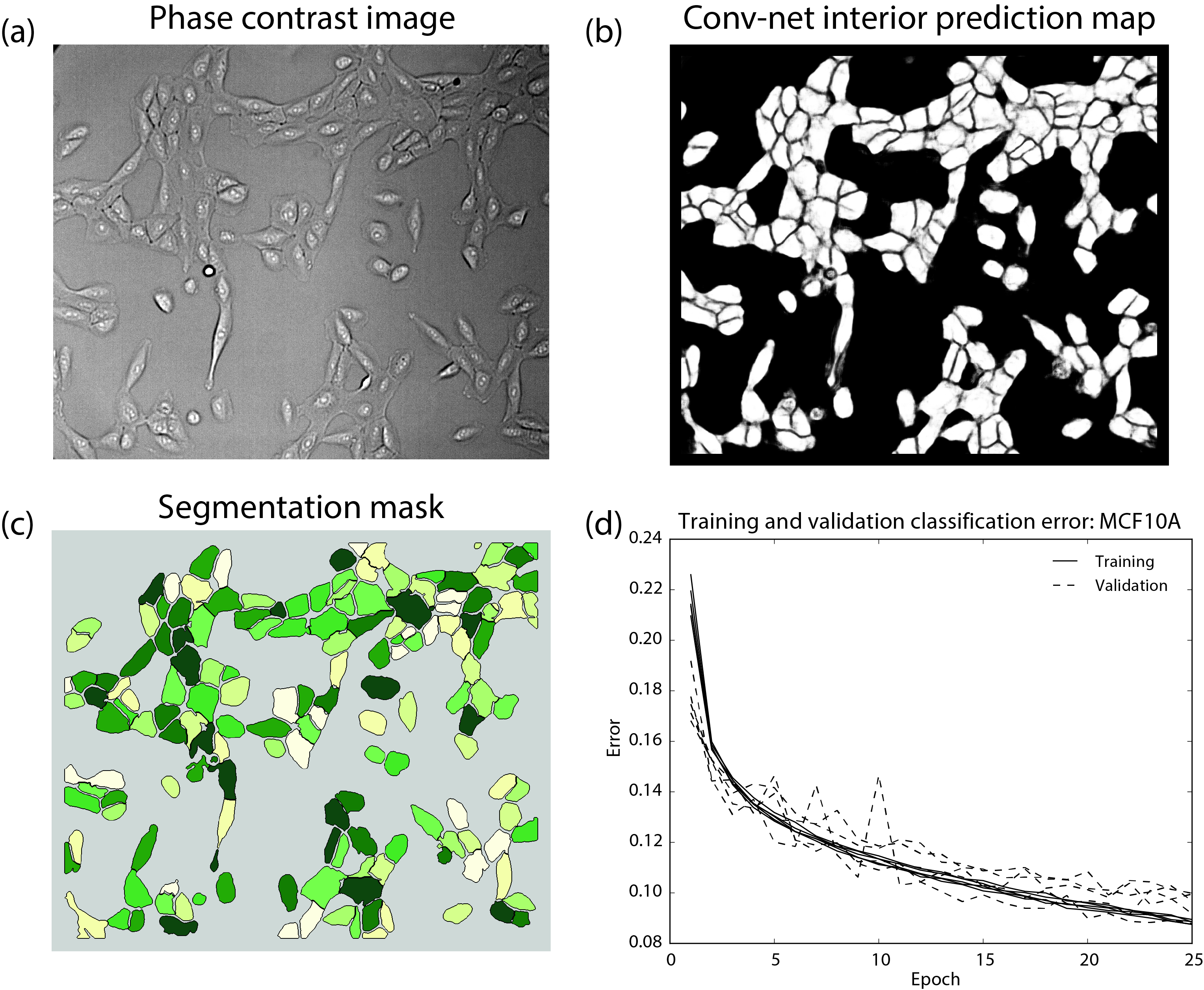

Supplement: S2 Fig — (TIF) [file pcbi.1005177.s003.tif]

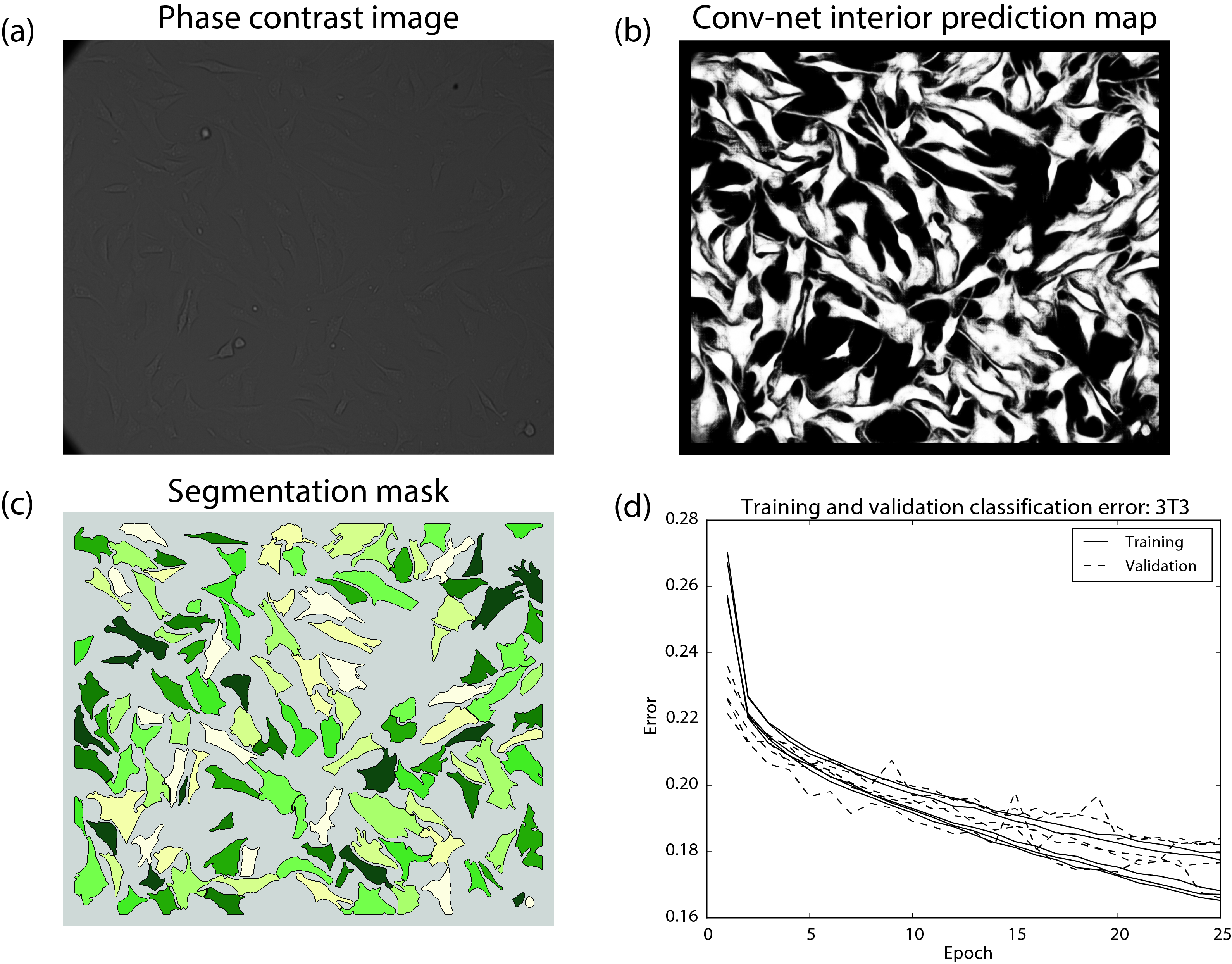

Supplement: S3 Fig — (TIF) [file pcbi.1005177.s004.tif]

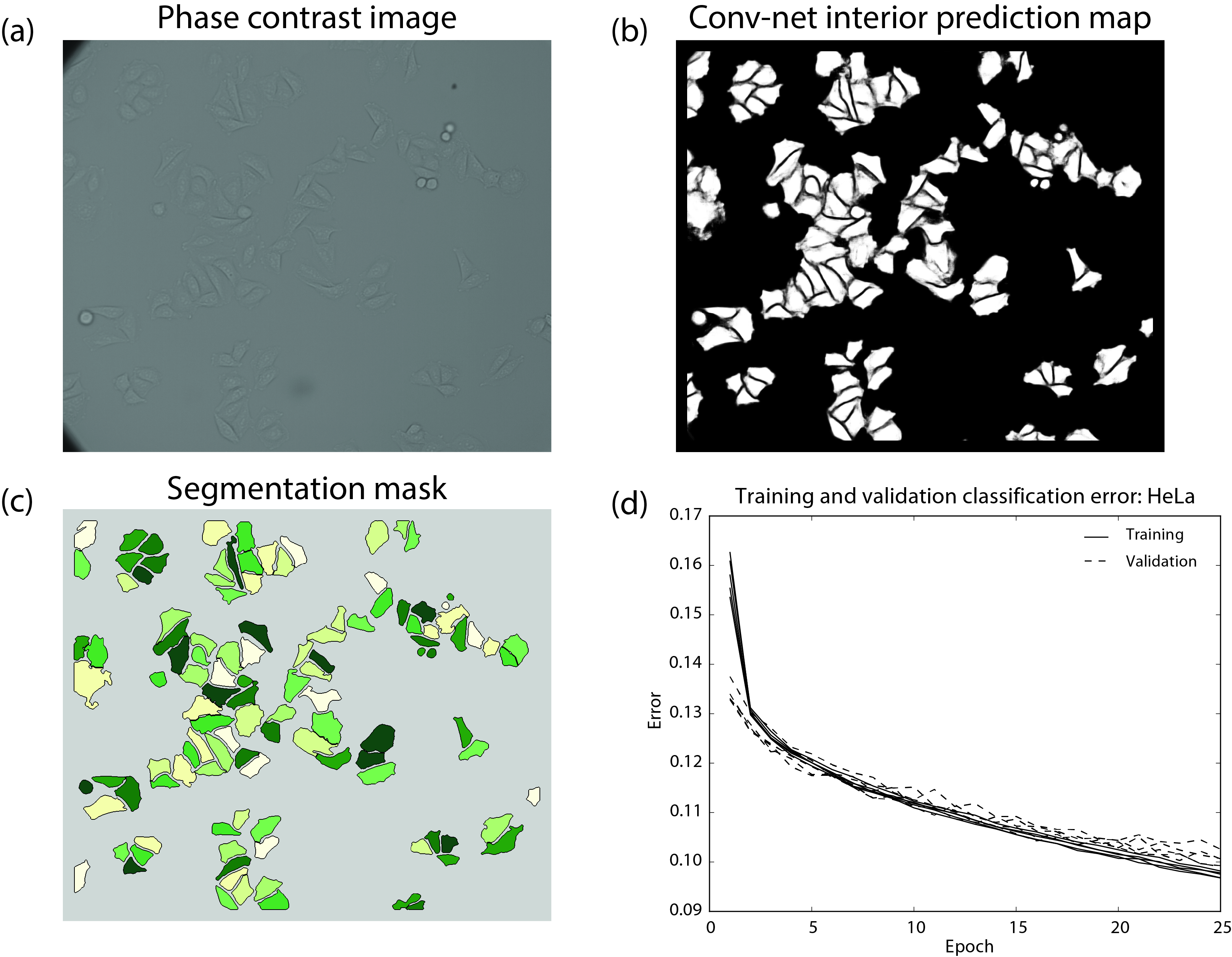

Supplement: S4 Fig — (TIF) [file pcbi.1005177.s005.tif]

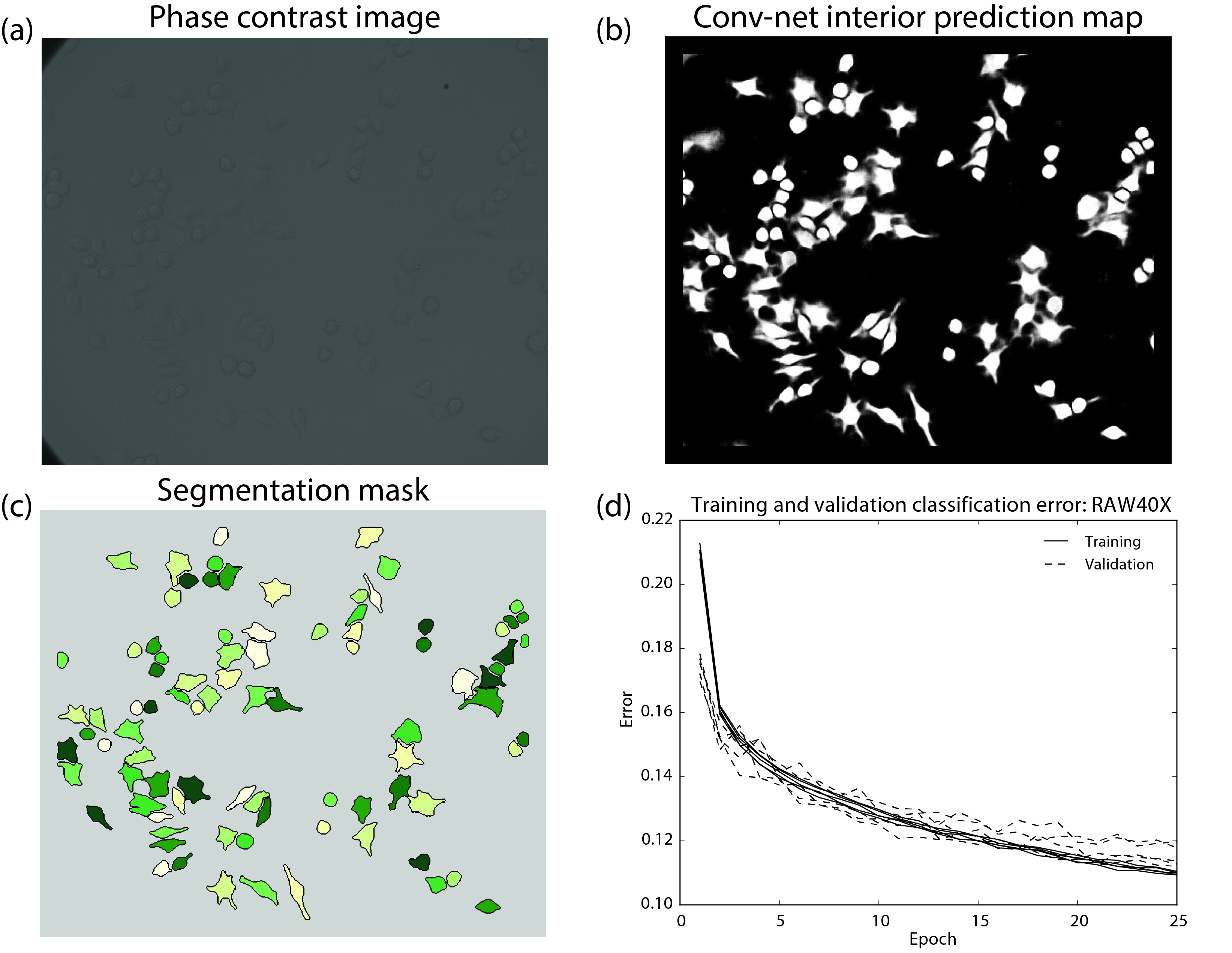

Supplement: S5 Fig — (TIF) [file pcbi.1005177.s006.tif]

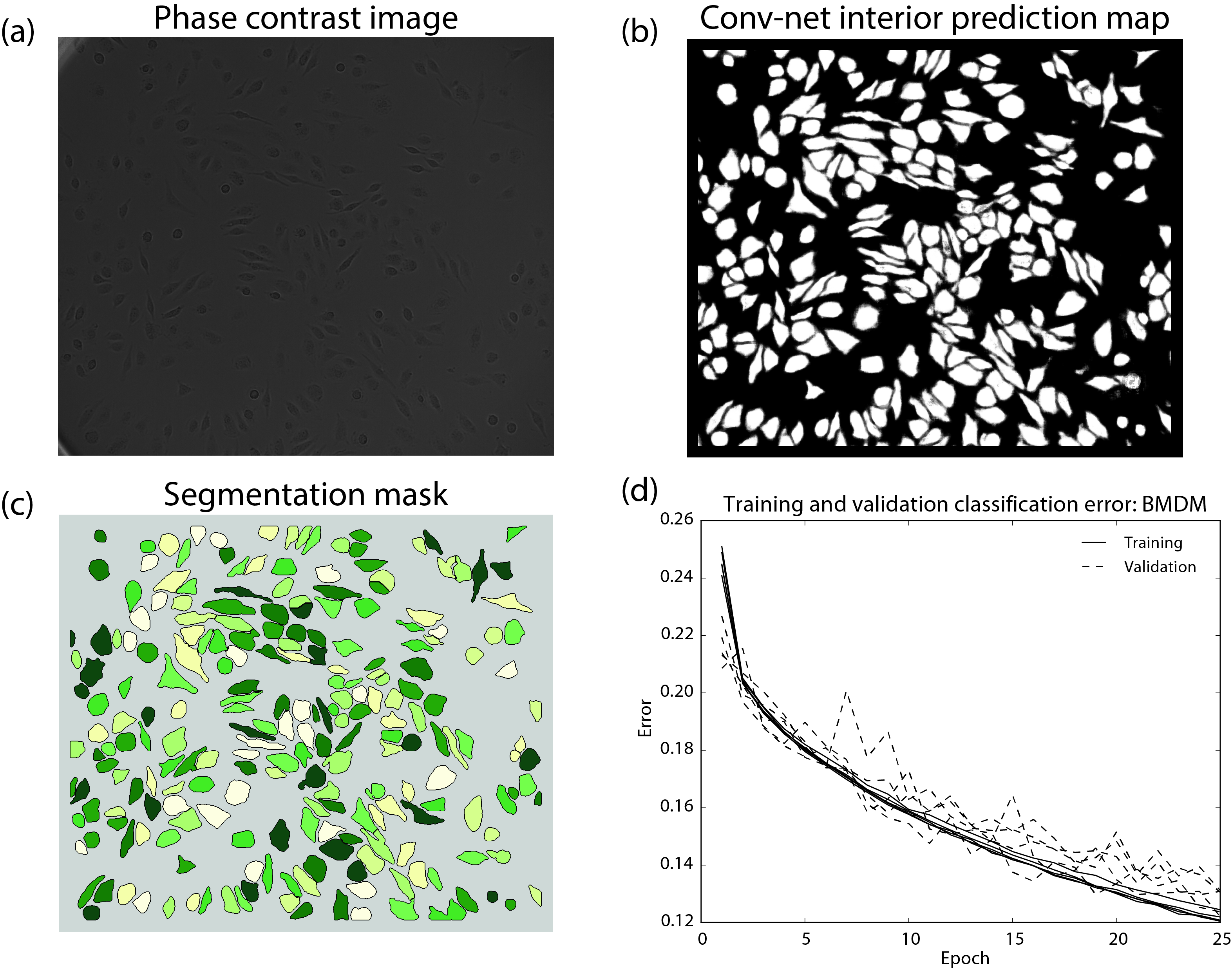

Supplement: S6 Fig — (TIF) [file pcbi.1005177.s007.tif]

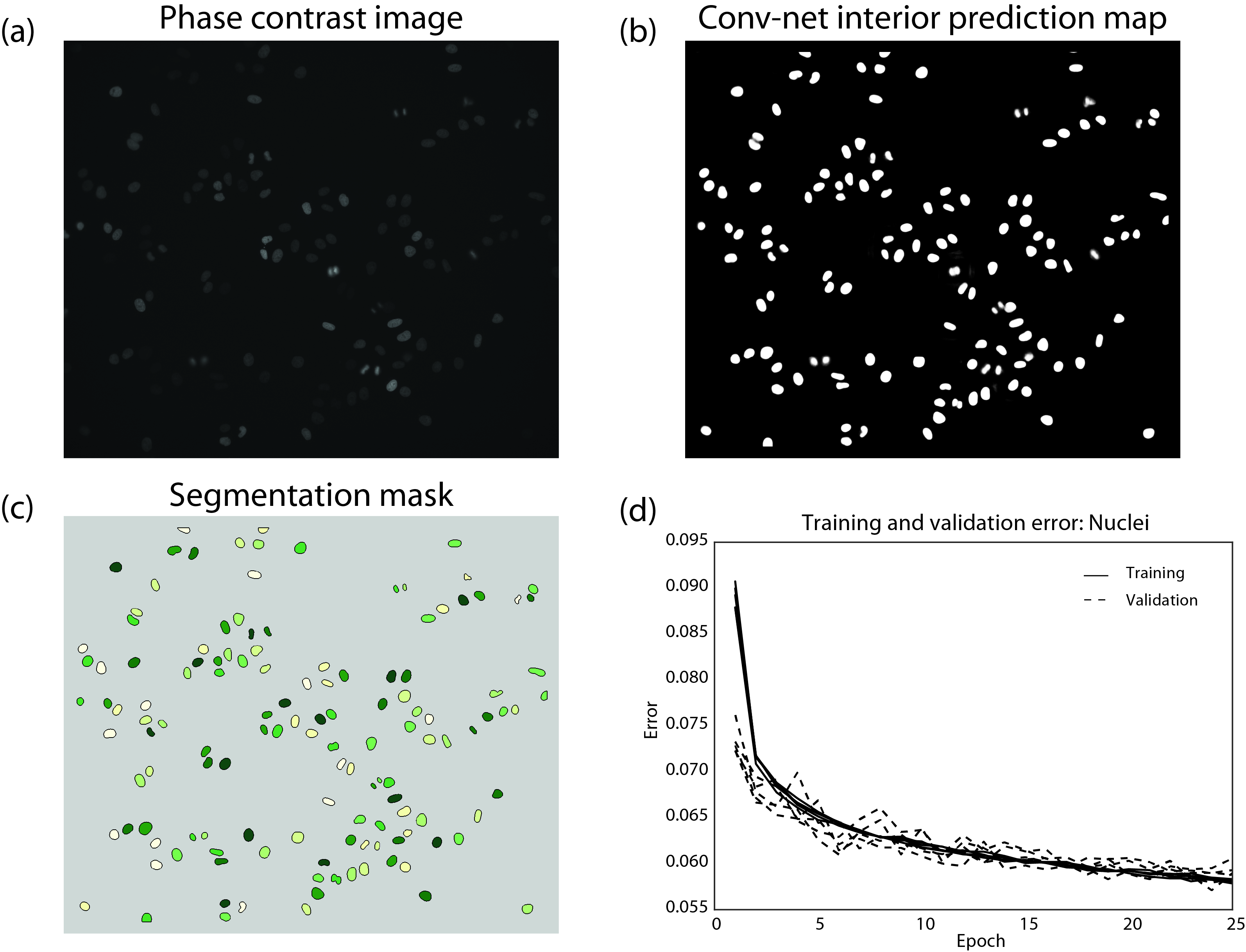

Supplement: S7 Fig — (TIF) [file pcbi.1005177.s008.tif]

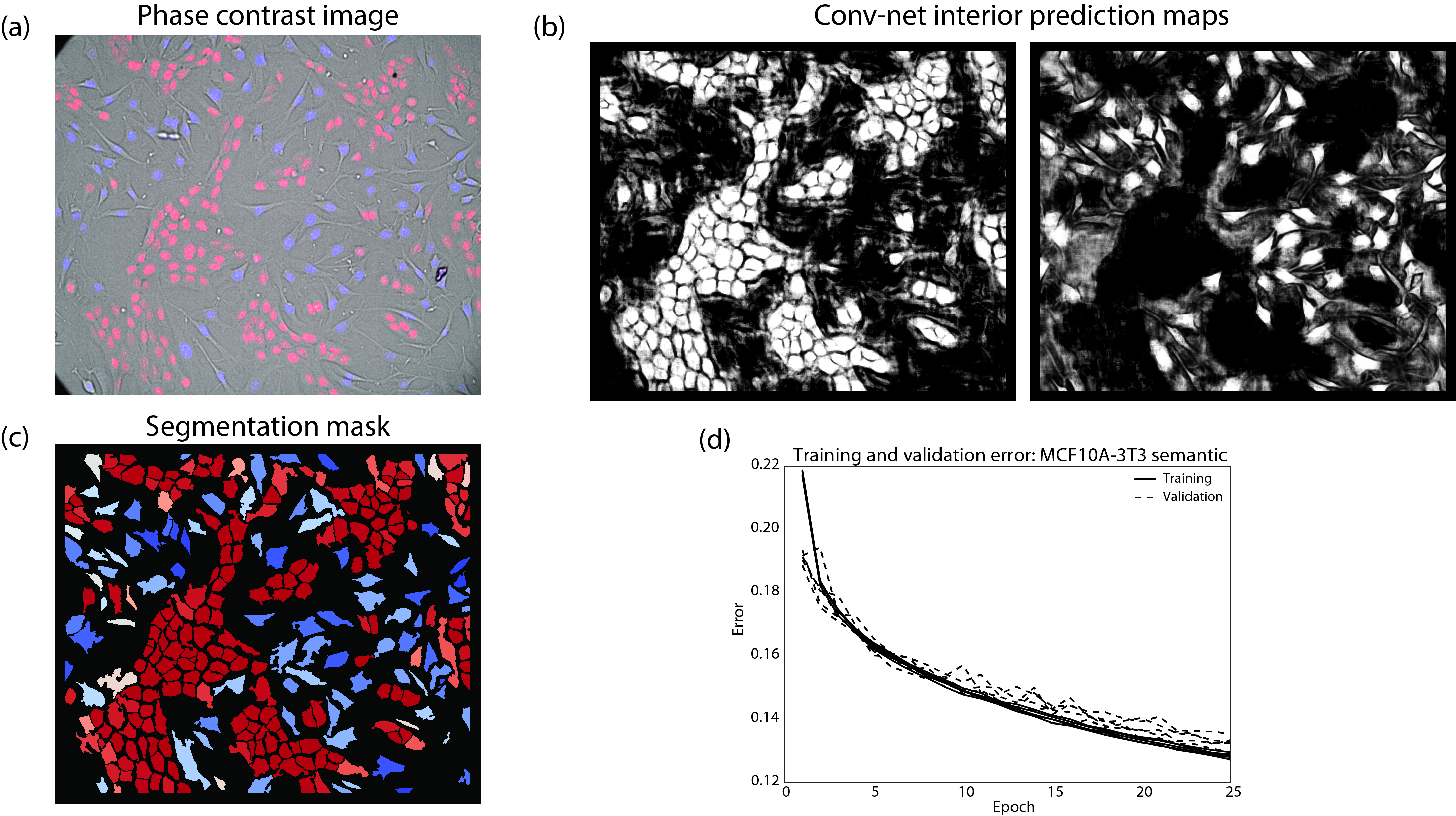

Supplement: S8 Fig — (TIF) [file pcbi.1005177.s009.tif]

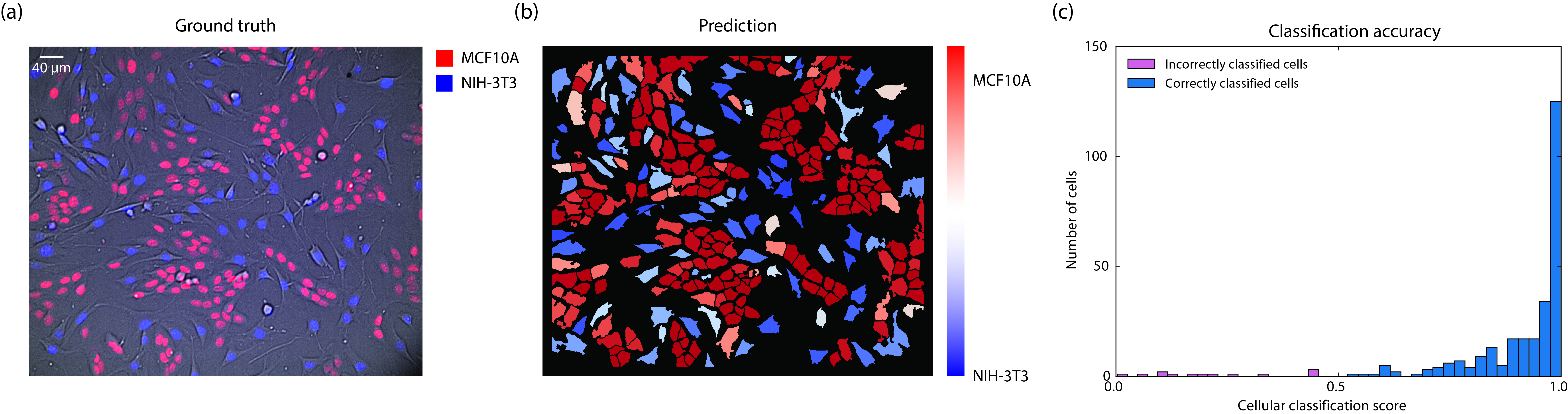

Supplement: S9 Fig — 286 cells were analyzed, including 93 3T3 cells and 192 MCF 10A cells. The classification accuracy was 89% for NIH-3T3 cells and 98% for MCF10A cells. (TIF) [file pcbi.1005177.s010.tif]

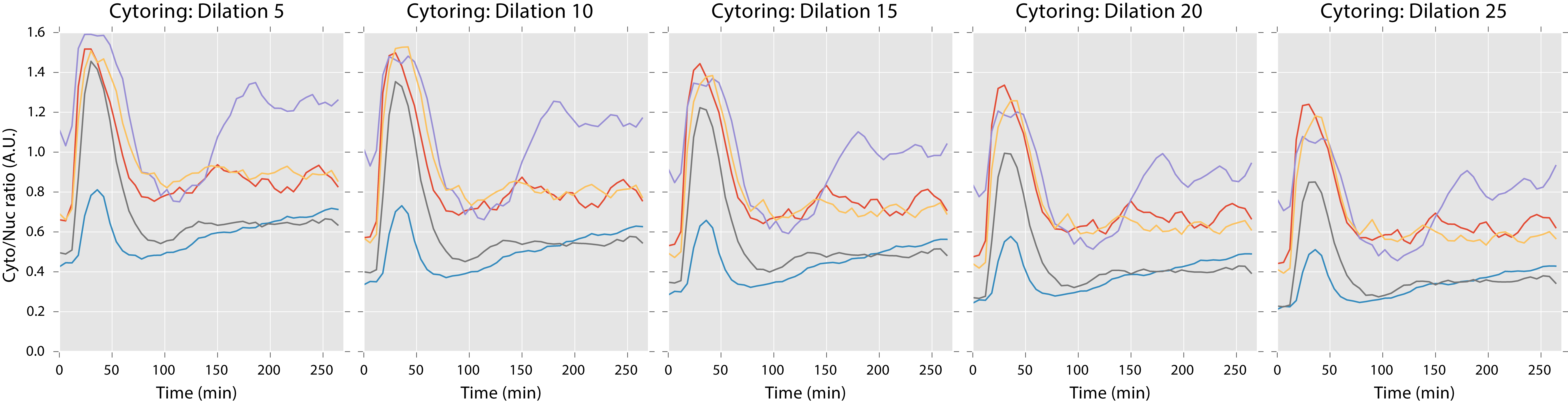

Supplement: S10 Fig — While the qualitative shapes of the curves remain intact as the size of the cytoring increases, there are important quantitative differences that emerge as the cytoring increases. In the grey trace, the first peak is identified as being quantitatively identical to the other cells in the plot if a 5 pixel cytoring is used, but becomes separated from the rest as the cytoring size is increased. A similar quantitative change can be seen in the size of the second peak in the purple trace, as it quantitatively becomes more diminished as the cytoring size is increased. (TIF) [file pcbi.1005177.s011.tif]

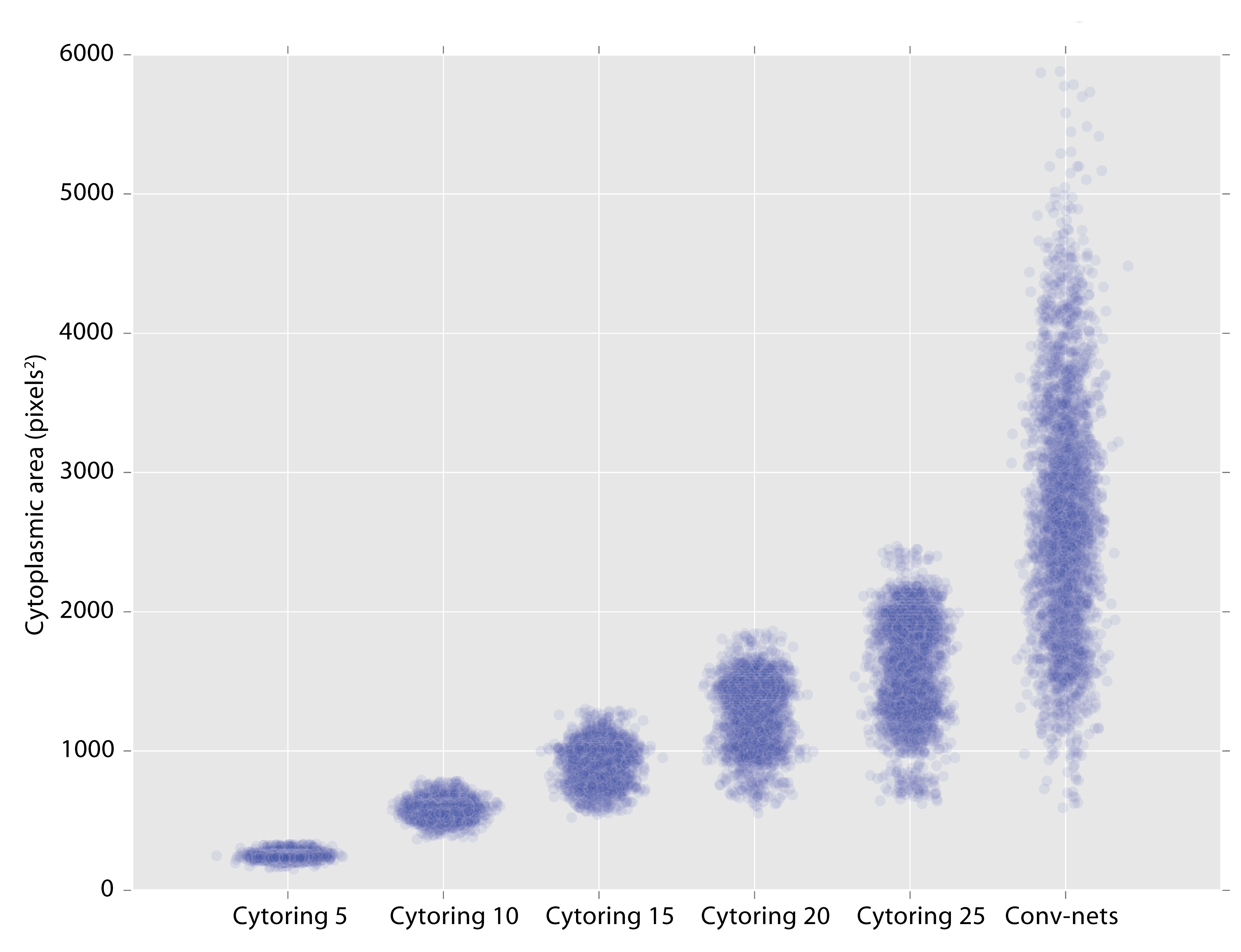

Supplement: S11 Fig — (TIF) [file pcbi.1005177.s012.tif]

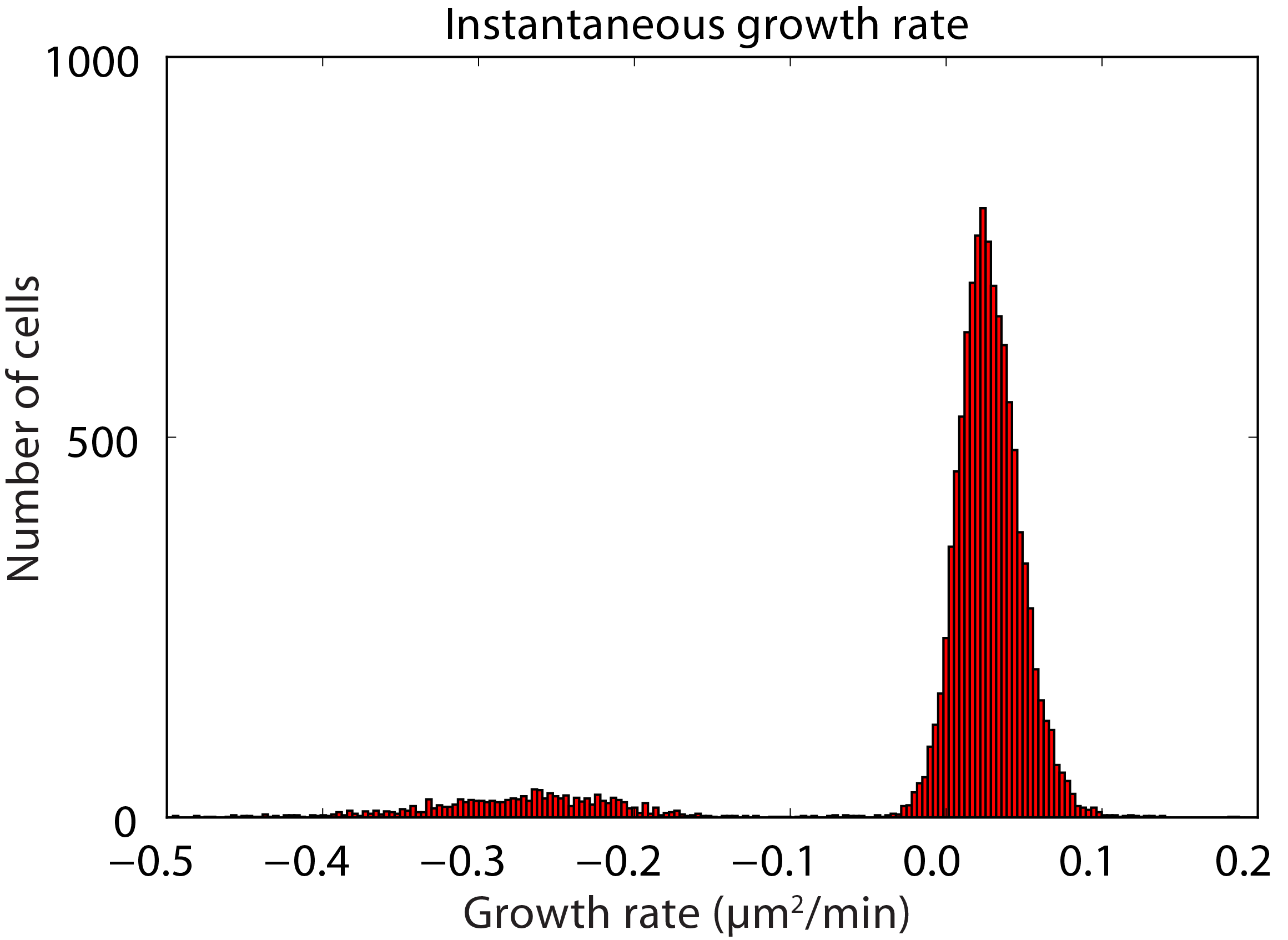

Supplement: S12 Fig — This histogram is identical to the histogram showed in Fig 3, with the axes expanded to show the negative growth rates corresponding to cell division. (TIF) [file pcbi.1005177.s013.tif]

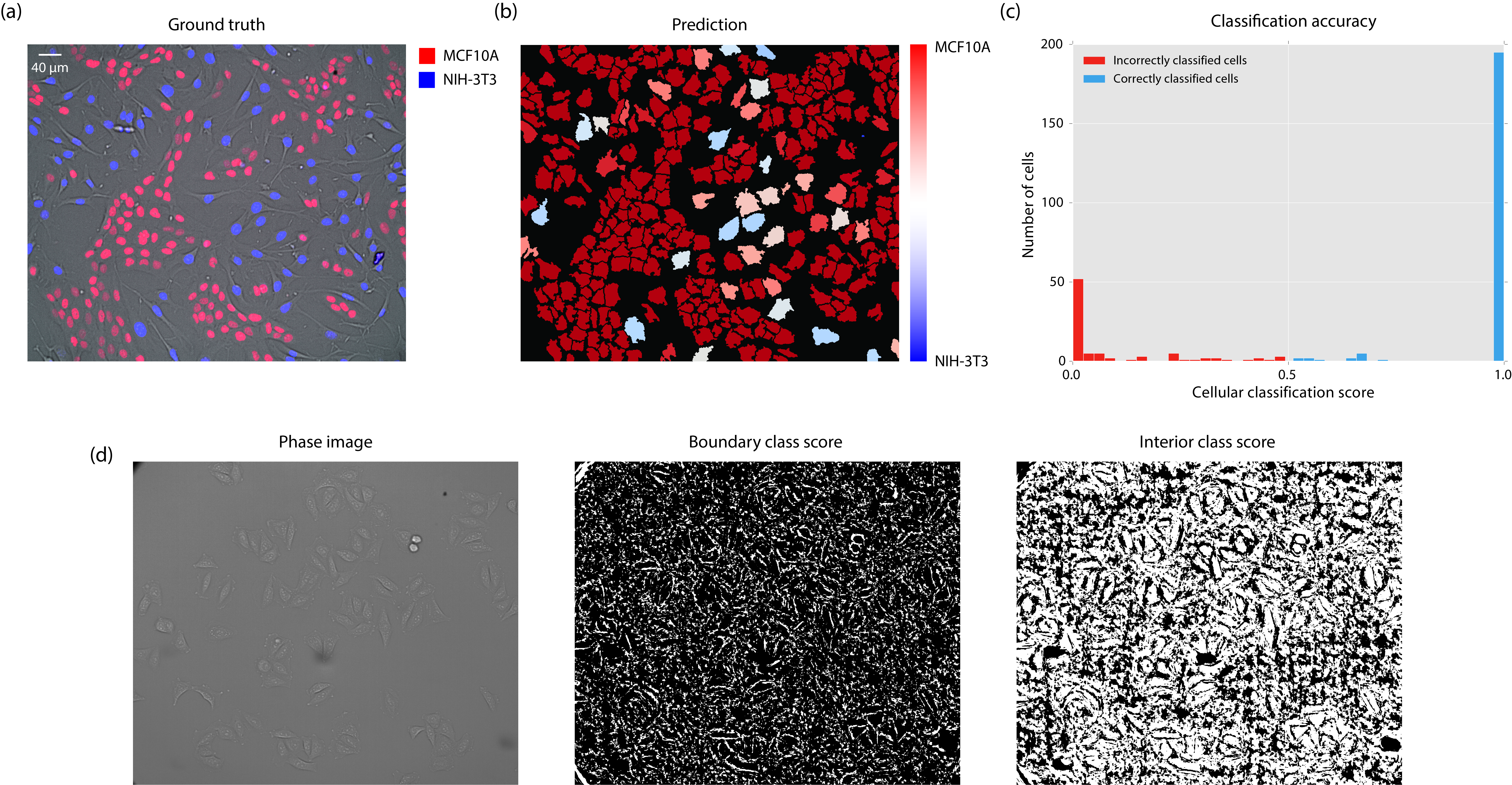

Supplement: S13 Fig — This figure highlights the importance of image normalization and receptive field size in training robust conv-nets. (a), (b), and (c). Mammalian-net semantic was trained on an un-normalized images of NIH-3T3 and MCF10A cells. Instead of learning differences in cell shape, the conv-net learned the brightness difference between the two dataset sources, leading to poor performance on images of co-cultures. 100% of MCF10A cells and 14% of 3T3 cells were classified correctly. (d) A conv-net with a 41x41 receptive field (as opposed to the 61x61 receptive field of mammalian net) was trained using a HeLa cell data set. Because of the smaller receptive field, the conv-net has difficulty distinguishing cell boundaries and interiors from background. (TIF) [file pcbi.1005177.s014.tif]

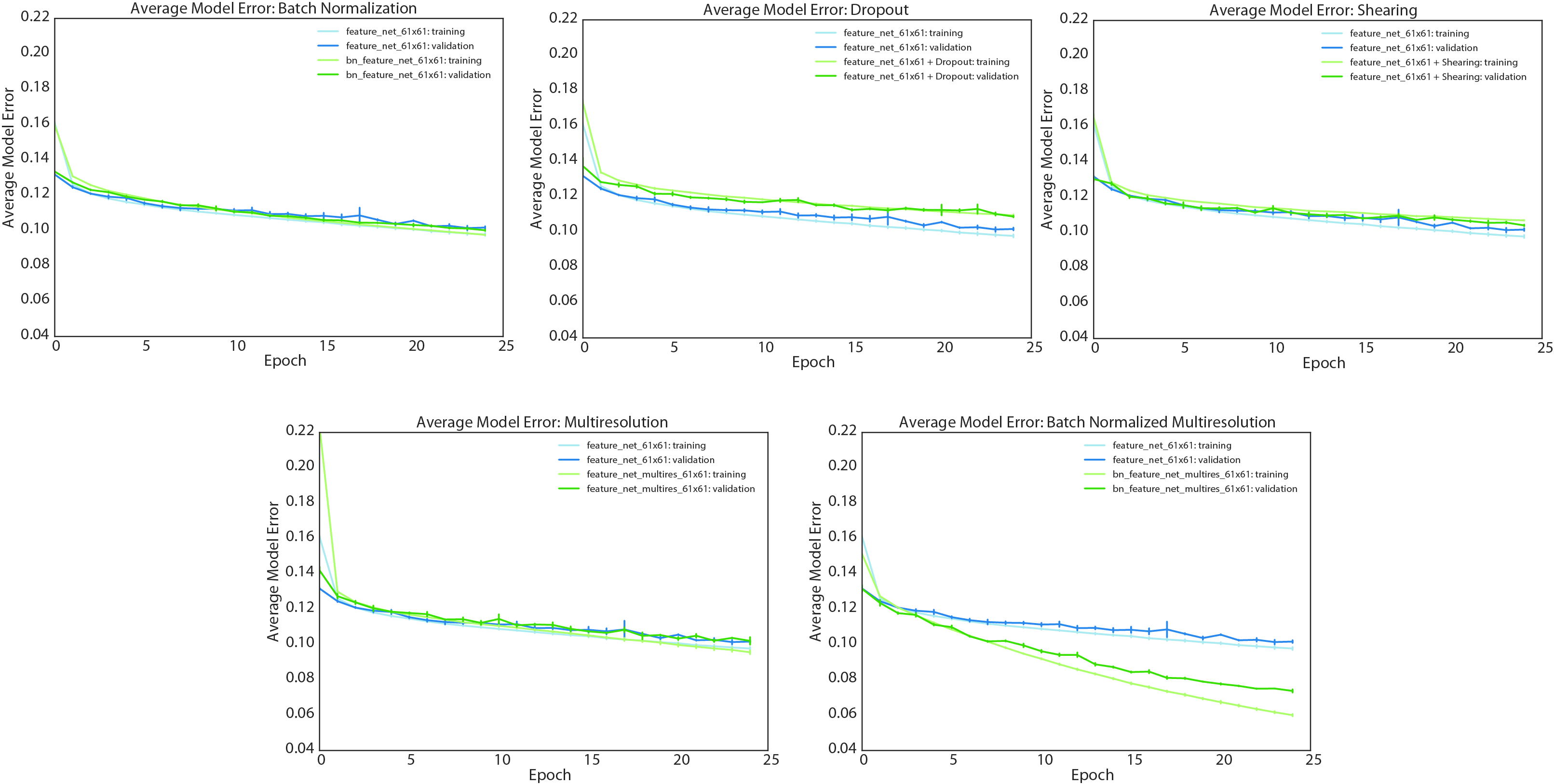

Supplement: S14 Fig — Dropout was only used in fully connected layers. The segmentation performance of each network as quantified by the Jaccard and Dice indices is provided in Table B in S1 Text. (TIF) [file pcbi.1005177.s015.tif]

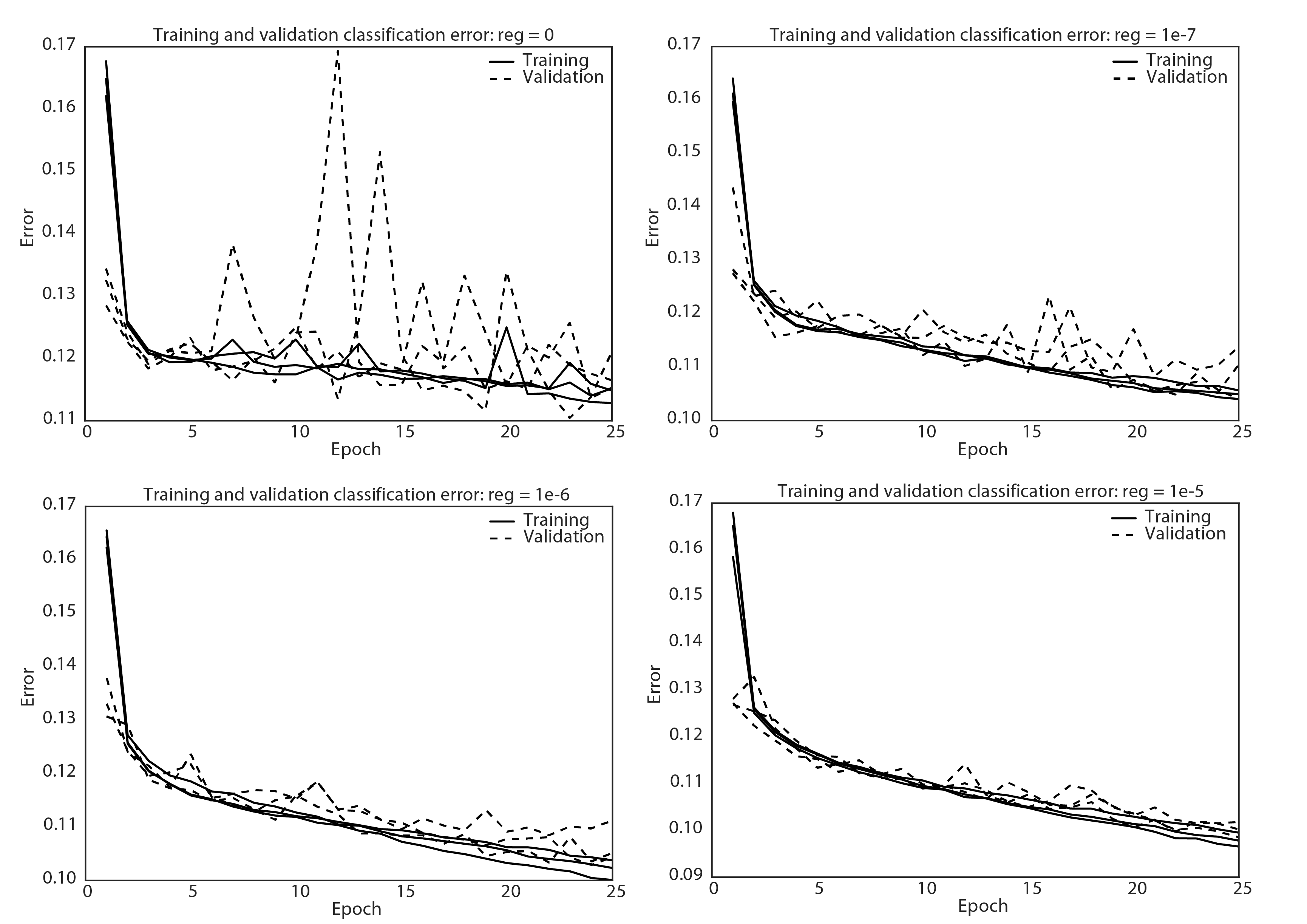

Supplement: S15 Fig — The L2 regularization parameter was varied from 0, 10−7, 10−6, and 10−5. Lower regularization was associated with more fluctuations in the classification error on the validation data set. (TIF) [file pcbi.1005177.s016.tif]

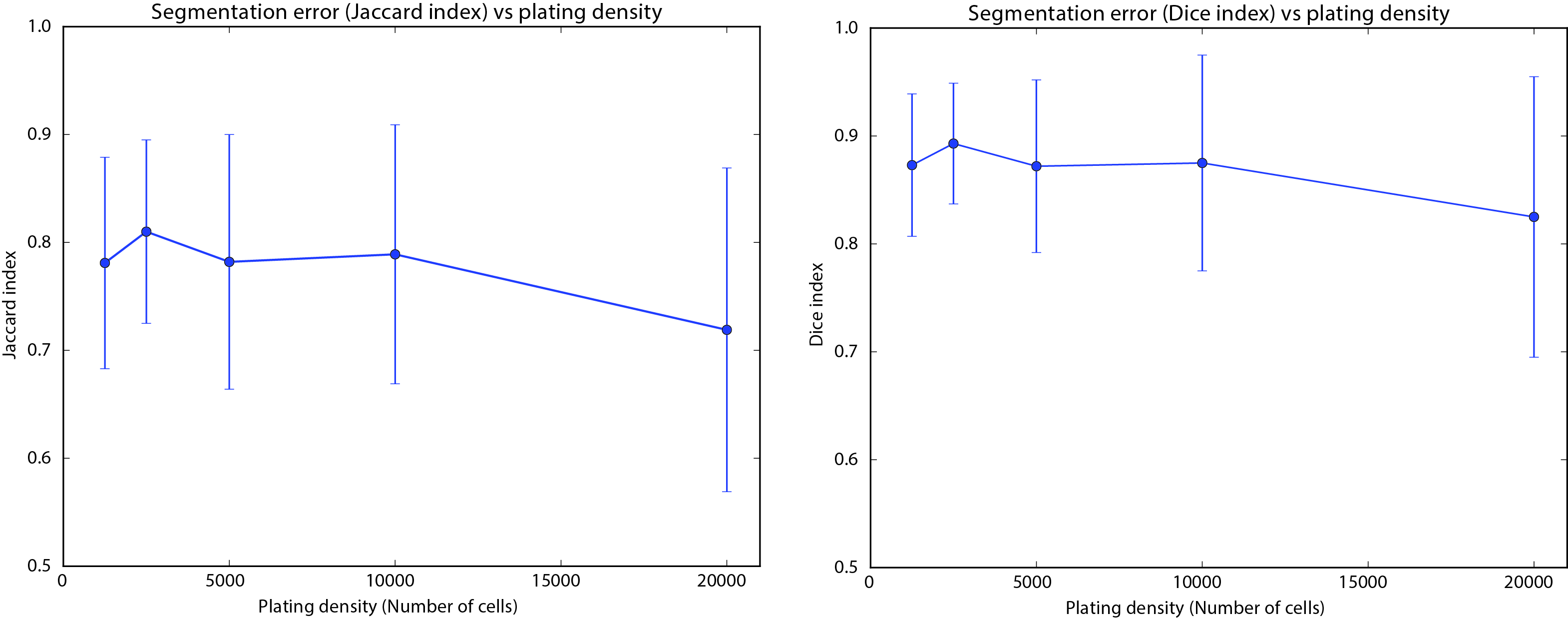

Supplement: S16 Fig — 1250, 2500, 5000, 10000, and 20000 cells were plated in the wells of a 96 well dish and imaged. The images were segmented manually and with conv-nets to compute the Jaccard and Dice indices. Segmentation performance remains stable for most plating densities, but decreases once cells are confluent. (TIF) [file pcbi.1005177.s017.tif]

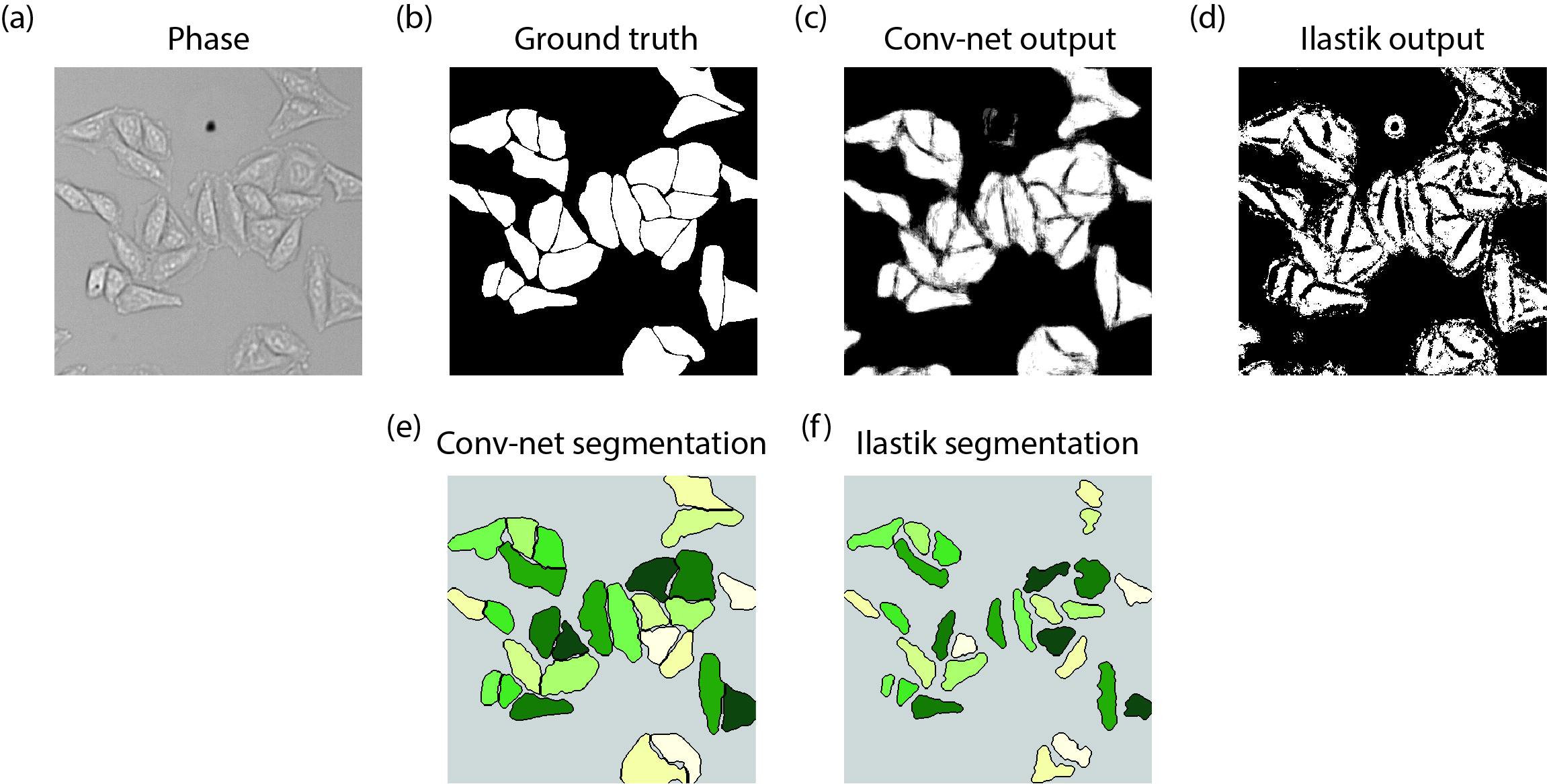

Supplement: S17 Fig — (a) Phase image of HeLa cells. (b) Ground truth for the cell boundary. (c) Conv-net soft-max score for edge and interior prediction. (d) Ilastik segmentation. (e) Conv-net segmentation after active contour processing. (f) Ilastik segmentation after active contour processing. (TIF) [file pcbi.1005177.s018.tif]
